# The *Drosophila* Midkine/Pleiotrophin Homologues Miple1 and Miple2 Affect Adult Lifespan but Are Dispensable for Alk Signaling during Embryonic Gut Formation

**DOI:** 10.1371/journal.pone.0112250

**Published:** 2014-11-07

**Authors:** Fredrik Hugosson, Camilla Sjögren, Anna Birve, Ludmilla Hedlund, Therese Eriksson, Ruth H. Palmer

**Affiliations:** 1 Department of Molecular Biology, Umeå University, Umeå, Sweden; 2 Department of Medical Biochemistry and Cell Biology, University of Gothenburg, Göteborg, Sweden; National Cancer Institute, United States of America

## Abstract

Midkine (MDK) and Pleiotrophin (PTN) are small heparin-binding cytokines with closely related structures. The *Drosophila* genome harbours two genes encoding members of the MDK/PTN family of proteins, known as *miple1* and *miple2*. We have investigated the role of Miple proteins *in vivo*, in particular with regard to their proposed role as ligands for the Alk receptor tyrosine kinase (RTK). Here we show that Miple proteins are neither required to drive Alk signaling during *Drosophila* embryogenesis, nor are they essential for development in the fruit fly. Additionally we show that neither MDK nor PTN can activate hALK *in vivo* when ectopically co-expressed in the fly. In conclusion, our data suggest that Alk is not activated by MDK/PTN related growth factors Miple1 and Miple 2 *in vivo*.

## Introduction

Since their identification the small secreted molecules Midkine (MDK) and Pleiotrophin (PTN) have been implicated in a multitude of developmental events including enhancement of cell growth and survival, cell migration, angiogenesis, neurite outgrowth and development (reviewed in [1], and [2]). MDK, also known as Retinoic acid-Inducible Heparin Protein (RIHP) [3], was first identified as a gene induced by retinoid acid in mouse embryo carcinoma cells [4], [5] and in an independent study a chicken homologue was purified from basement membranes [6]. The related family member PTN also known as Heparin-Binding Growth-Associated Molecule (HB-GAM) [6]–[8], Heparin-Binding Neurotrophic Factor or Neurite-promoting Factor (HBNF) [9], Osteoblast Specific Protein-1 (OSF-1) [10] and Heparin Affin Regulatory Peptide (HARP) [11], was first purified from bovine uterus as a weak mitogen towards fibroblasts. Given that both MDK and PTN encode small secreted cytokines with heparin-binding properties, they have long been considered to be functional ligands for cell surface receptors.

A number of reports have specifically addressed the role of MDK and PTN signaling through the Anaplastic Lymphoma Kinase (ALK) receptor tyrosine kinase (RTK) [12], [13]. In these studies it has been reported that activation of ALK by either MDK or PTN leads to activation of both ERK and PI3K pathways [14]–[17]. However, a number of independent studies present contradictory results showing that MDK and PTN do not activate ALK [18]–[21].

To date, homologues have been reported in most species within the chordate and arthropod phyla, including human, mouse, fish, chicken, frogs and insects [3], [6], [7], [22]–[25]. In *Drosophila melanogaster* the *miple1* (also referred to as *miple*) and *miple2* genes display significant homology to the vertebrate MDK/PTN family [25]. In *Drosophila*, the expression of *miple1* and *miple2* is dynamic throughout embryogenesis, with both genes expressed in spatially and temporally restricted patterns. The transcript of *miple1* is mainly expressed in the developing CNS, while *miple2* transcripts are present in a number of cells within the developing endoderm [25]. Until now, no genetic characterisation of these two genes has been accomplished and their functional importance has therefore remained elusive.

In *Drosophila melanogaster* the Alk RTK together with its ligand Jelly Belly (Jeb) plays a well described critical role in the development of the embryonic visceral musculature [26]–[29]. Alk is expressed in progenitors of the developing visceral mesoderm (VM) where it is activated by the LDL domain containing ligand Jeb, leading to activation of the MAPK signaling pathway in a restricted subset of VM cells that subsequently differentiate into muscle founder cells (FCs). Alk activation by a Jeb-like ligand is functionally conserved in the nematode *C. elegans* where an Alk homologue is encoded by the *scd-2* gene [30], [31] and the Jeb homologue *hen-1* [32]. Together they regulate dauer formation in the worm by modulating TGF-β signaling and its response to dauer pheromone [33].

In addition to their role in the specification of FCs during development of embryonic visceral mesoderm, signaling mediated by the Jeb/Alk ligand-receptor pair is employed in photoreceptor axonal targeting during the late maturation of the optic lobe neuropile [34], [35]. Additionally, Jeb/Alk signaling regulates development and neuronal transmission of the larvae neuromuscular junction (NMJ) [36], [37] and is required to protect larval neuroblasts during starvation in a process similar to brain sparing [38]. Alk is also involved in body size regulation, memory and learning [39], [40] and ethanol sensitivity [41].

While the mechanisms by which MDK/PTN mediate signaling are fundamental for understanding their role in disease as well as development, their involvement in signaling and their receptor specificity *in vivo* are poorly understood. *Drosophila* has two well conserved homologues of the MDK/PTN family, Miple1 and Miple2 in addition to the characterised Alk ligand Jeb. Therefore the fruit fly presents a unique opportunity to study and characterise their function with focus on their potential interactions with Alk. In this work we have deleted the loci encoding the two *Drosophila* MDK/PTN family members: *miple1* and *miple2*, each individually and together. We conclude that *miple1* and *miple2* are not essential for normal development, since both single and double mutant flies are viable and fertile. Furthermore, we have examined a potential relationship of the Miple proteins with the Alk signaling pathway. Neither Miple1 nor Miple2 are able to activate Alk or rescue phenotypes caused by the loss of the Alk ligand Jeb, nor are they able to ectopically activate Alk signaling in the eye. Taken together, our data suggests that neither Miple1 nor Miple2 activate the Alk RTK *in vivo* during *Drosophila* development.

## Materials and Methods

### Ethical statement

All rabbit immunisations were approved by the Umeå Ethical Board of Animal Research, Umeå, Sweden, according to the declaration of Helsinki (permit number A56-04).

### *Drosophila* maintenance and genetics

Standard *Drosophila* husbandry procedures were followed. *Drosophila* strains were maintained on potato-meal based medium, and raised at room temperature. All crosses involving Gal4/UAS system and parental crosses for experiments were performed at controlled 60% humidity conditions at 25 degrees. Fly strains used in this study: *P{EPgy2}^EY04126^* (Bloomington 15720) was used to generate *miple* deletions, *Df(3L)BSC125* (Bloomington 9290) is a deficiency uncovering both *miple* genes (in total eight genes and two small non coding RNAs), *Df(3L)BSC126* (Bloomington 9291) is a deficiency uncovering *miple2* (in total eight genes). Dr^Mio^/TMS, P{Δ2–3}99B (Bloomington 406) was employed as transposase source for the initial excision screen. *Blm* ^D3^ (Bloomington 8656) and *Blm* ^D2^ {Δ2–3}99B (Bloomington 8657) were employed in the second excision screen to induce larger deletions [42]. *jeb^weli^* [29], *Df(2R)BSC199* (Bloomington 9626) - a deficiency uncovering the *jeb* locus, *UAS-jeb* [43], *UAS-Alk* [44], *UAS-hALK^WT^ and UAS-hALK^F1174L^* [45] have been described previously. *miple* mutant alleles *miple1^Δ194^ miple2^Δ396^ miple1,miple2^Δ104^*, *control^rev657^*, *UAS-miple1*, *UAS-miple2-venus*, *UAS-MDK* and *UAS-PTN* were generated in this study. The following drivers were employed for over-expression experiments: *en-Gal4* (Bloomington 30557), *GMR*-*Gal4* (Bloomington 1104), *sevEP*-*Gal4* (Bloomington 5793), *twist2xPE*-*Gal4* (Bloomington 2517), *Actin5C*-Gal4 (Bloomington 4414), *Mef2-Gal4* (kind gift from B. Dickson), *C155-Gal4* (Bloomington 458), *MS1096-Gal4* (Bloomington 8860) and *da-Gal4* (Bloomington 8641). The *rP298-LacZ* [46] enhancer trap was used to identify muscle founder cells. For rescue experiments the following stocks were generated: *en-Gal4, jeb^weli^/CyO wg-LacZ*, *UAS-miple1*, *BSC199/CyO wg-LacZ*, *UAS-miple2venus/UAS-miple2venus*; *BSC199/CyO wg-LacZ* and *UAS-jeb, BSC199/CyO wg-LacZ*. For over-expression experiments *sevEP-Gal4, UAS-Alk/CyO* were generated. *PBac{3PHy+}C205* (Bloomington 1603) was identified as a putative *ttm2* mutant allele inserted upstream of *ttm2*. The *miple2-Gal4* [47], *miple1-Gal4 P{GMR54D02-GAL4}attP2* (Bloomington 45783 [48]), and *UAS-RedStinger* (Bloomington 8546) were used for expression analysis.

### Generation of *miple* mutants

Individual *miple1^Δ194^* and *miple2^Δ396^* mutants and the *control^rev657^* were generated by imprecise excision of *P{EPgy2}^EY04126^*. Double deficient *miple1,miple2^Δ104^* flies were generated by imprecise excision of *P{EPgy2}^EY04126^* in a *Blm^D2^/Blm^D3^* mutant background [42]. Deletion breakpoints in mutant flies were verified by DNA sequencing. Sequencing of *control^rev657^* confirms an intact sequence of both *miple* genomic loci after excision of *P{EPgy2}^EY04126^* and this stock as therefore was employed as control. *miple1^Δ194^* has a deletion covering 3L:266503-271594 and *miple2^Δ396^* 3L:271602-274471. The large deletion in *miple1,miple2^Δ104^* uncovers region 3L:267332-275960. Gene structure was generated using Fancygene [49].

### Generation of UAS-miple transgenes and expression vectors

To generate *UAS-miple1* transgenic flies, DNA encoding the open reading frame (ORF) of *miple1* was generated by PCR and subcloned into pUAST. *UAS-miple2-YFP.venus* and *UAS-miple2.6Xmyc* transgenic flies were generated by cloning of *miple2* from the RE73766 EST (DGRC) into pUAST-C-terminal Venus (pTWV, 1092, DGRC) and pUAST-C-terminal 6xMyc (pTWM, 1108, DGRC) respectively using Gateway Technology. *UAS-MDK* transgenes were generated by EcoRI and XhoI excision from the BC011704 (ATCC) cDNA clone, prior to ligation into pUAST vector. *UAS-PTN* was generated by PCR from the BC005916 (ATCC) cDNA clone using the primer pair 5′-TATGAATTCTTGCAACAAAGGCAG-3′ and 5′-ATACTCGAGTATAAGCCCCTACTGG-3′, followed by cloning into pUAST using EcoRI and XhoI. Constructs were confirmed by DNA sequencing and used for generation of transgenic fly strains (BestGene Inc.). The pcDNA3-*MDK*, and pcDNA3-*PTN* constructs was generated as described above.

### Heparin binding assay

Heparin binding assays were performed as described in [50]. Briefly, pIRES-*miple2*, pcDNA3-*miple1*, pcDNA3-*MDK*, and pcDNA3-*PTN* (this study) were transfected into HEK293 cells, and conditioned medium was collected after 24 hrs, incubated overnight at 4°C with heparin-agarose (Sigma) and pre-washed with equilibration buffer (20 mM Tris pH 7.5). Beads were subjected to washing 5 times with washing buffer (50 mM NaCl, 20 mM Tris pH 7.5) prior to elution in elution buffer (0.9 M NaCl, 20 mM Tris pH 7.5). Samples of 20 µl for both input and output were separated by SDS-page and detected by immunoblotting using anti-HA monoclonal antibody 1∶1000 (Covance), guinea pig anti-Miple1 1∶1000 [25], rabbit anti-Miple2 1∶500 (this study), rabbit anti-Midkine 1∶500 (Abcam) and goat anti-Pleiotrophin 1∶500 (R&D Systems), visualised with secondary antibodies mouse-HRP 1∶4000 (GE Healthcare), guinea pig-HRP 1∶5000 (Jackson), rabbit-HRP 1∶10000 (GE Healthcare) sheep-HRP 1∶3000 (Pierce) using the LiCor Odyssey system.

### Generation of Miple2 antisera

For preparation of Miple2 antisera, DNA encoding amino acids 21-279 of Miple2 was subcloned into the pGEX-T2 GST fusion protein expression vector. The reading frame of the GST-Miple2 sequence was subsequently analysed by DNA sequencing. GST-Miple2 fusion protein was induced and purified from *E. coli* (BL21(DE3)) bacterial lysates by standard protocols using glutathione sepharose beads (GE Healthcare). GST-Miple2 recombinant protein was proteolytically cleaved with thrombin (4 units/ml). After cleavage, thrombin was removed with p-aminobezamidine agarose and the sample dialysed overnight, with a cut-off of 15 kDa, followed by concentration. The resulting Miple2 recombinant protein was used for immunisation of rabbits (Agrisera AB, Vännäs, Sweden). Antisera was IgG purified (PIERCE Biotechnology) and subsequently affinity purified by standard protocols using glutathione sepharose beads. The previously generated Miple1 antisera [25] was IgG purified (PIERCE Biotechnology) and then affinity purified by standard protocols using Ni-NTA beads (Qiagen).

### Immunohistochemistry and Western Blot

Embryo staging was carried out according to [51]. Embryos were collected and fixed according to standard protocols except for dpERK staining which require fixation in 8% formaldehyde followed by incubation with the following primary antibodies, rabbit anti-β-galactosidase 1∶200 (Chappel), guinea pig anti-Alk 1∶1000 [27], mouse anti-dpERK 1∶500 (Sigma), rat anti-Org-1 1∶100 [52], guinea pig anti-Vrp1 1∶1000 [53], mouse anti-Fasciclin III 1∶50 mAb-7G10 (DSHB). Larvae tissues were dissected in ice cold PBS and fixed in 4% formaldehyde in PBS, 0,1% Triton for 30 minutes in RT or at 4 degrees overnight. Tissues were stained with DAPI 1∶1000 (1 mg/ml, Invitrogen) for 5 minutes to detect nuclei. Larvae eye discs were stained with rabbit anti-MDK 1∶1000 (Abcam) and goat anti-PTN 1∶1000 (R&D Systems). Secondary fluorescent antibodies from Jackson and Amersham were employed for detection. Images of embryos were acquired using a Leica SP2 confocal and LAS AF software, and larval eye discs images acquired with a Zeiss Axio Imager.Z2 microscope and Axio vision Release 4.8 software, prior to assembly with Adobe Photoshop CS5. Western blot was performed using standard protocols. Samples were prepared from adult fly heads, and 50 µg of total protein was used for expression analysis. Protein was detected using guinea pig anti-Miple1 1∶1000 [25] and mouse anti-α-tubulin 1∶10000 (T5168 Sigma) visualised with secondary antibodies guinea pig-HRP 1∶5000 (Jackson), mouse-HRP 1∶4000 (GE Healthcare) using the LiCor Odyssey system.

### Survival analysis

Homozygous virgin females of indicated genotype were crossed to *Df(3L)BSC125/TM3 actinGFP* males in vials and transferred to new vials every 24 hours. After scoring genotype and sex of hatched adult progeny, the average percentage between homozygous (mutant or control over deficiency) and heterozygous (mutant over balancer), was calculated from three biological replicates.

### Electron & Stereo microscopy

Adult flies were fixed in 2.5% formaldehyde, 2.5% glutaraldehyde in PBS before standard processing for Scanning Electron Microscopy (SEM). For light imaging a Nikon SMZ150 stereo microscope was employed. To generate a merged z-stacked image the EDF module in the NIS-BR software (Nikon) was used.

### Pupal size measurements

Parental flies were kept in bottles for at least two generations and subsequently used for crosses of 10 males and 10 females in vials. Dark pupae were scored by absence of sex combs and sex was further confirmed after hatching. Sample pupae were aligned on sticky tape and measured using the Zeiss Axio Imager.Z2 microscope and Axio vision Release 4.8 software.

### Life span and fecundity measurements

Parental flies were kept in bottles for at least two generations and then used for crosses (>90 for over-expression experiments and >113 for mutant experiments). Flies of age 1–5 days were used for lifespan experiments performed in an incubator with controlled conditions of light/dark cycles, humidity and temperature (12 h/12 h, 60% and 25 degrees). 25 flies/vial of the same gender were transferred to new food vial every 2–3 days and dead flies were counted. Fecundity experiments were modified from [54] and [55]. 1–2 days old females were mated to *w^1118^* males until age 4–5 days, after which males were removed and the number of embryos produced by females during each 24 hour period were analysed for 5 days.

### *In situ* hybridisation

The entire coding regions of *miple1* and *miple2* were cloned into the dual PCR II promoter TOPO vector (Invitrogen) and used as template to generate DIG-labeled anti-sense and sense probes using SP6/T7 RNA polymerase (Roche) and NTP/DIG-UTP mix (Roche). *In situ* hybridisation of larval tissues was performed according to [56], while for adult brains a protocol including Proteinase K treatment was employed [57]. Samples were mounted on polylysine coated slides and visualised using DIC with Zeiss Axio Imager.Z2 microscope and Axio vision Release 4.8 software.

## Results

### *miple1* and *miple2* encode small secreted heparin binding proteins that are dispensable for Drosophila development

**Figure 1:**
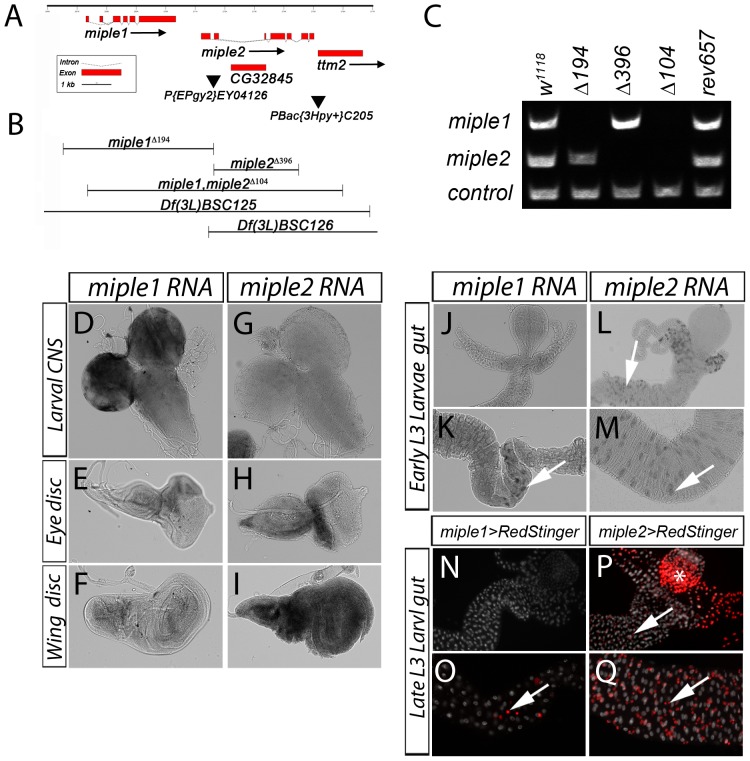
Generation of *miple* deletions and larval expression of *miple1* and *miple2*. (**A**) Schematic of the chromosomal region comprising the *miple1* and *miple*2 loci on 3L with exons represented by boxes (translated regions depicted as broad red boxes; untranslated are dotted lines). The P-element employed for the imprecise excision screen, *P{EPgy2}EY04126*, is shown as filled inverted triangle. In addition to *miple1* and *miple2*, two other genes – *CG32845* (within the first intron of *miple2*) and *ttm2* (downstream of *miple2*) are shown (red boxes). A putative *ttm2* allele caused by the P-element *PBac{3Hpy^+^}C205* upstream of *ttm2* is shown as filled inverted triangle. (**B**) Regions deleted in the *miple1^Δ194^ and miple2^Δ396^* single, and the *miple1,miple2^Δ104^* double mutants are shown. Also indicated are the deletions in the deficiency lines *Df(3L)BSC125* and *Df(3L)BSC126.* (**C**) Genomic PCR confirming gene deletion of *miple1* and *miple2* in the single and double mutants as indicated, controls are *w^1118^* and revertant *control^rev657^*. The control (lower band) was carried out to confirm genomic DNA quality. (**D-I**) *In situ* of *miple1* and *miple2* mRNA in larval tissues. Strong expression is detected in the larval optic lobe and CNS (D) and weak expression in imaginal eye (E) and wing discs (F). *miple2* mRNA is not detectable in the larval CNS (G), while imaginal discs exhibit strong expression, represented by eye (H) and wing discs (I). (**J-M**) In early L3 larvae *miple1* mRNA expression is detected in a distinct population of cells in the anterior midgut (K, see arrow) but not detected in other parts of the anterior midgut (J). Expression of *miple2* mRNA expression is also detected in early L3 larvae in cells of the anterior midgut (L, see arrow) and posterior midgut (M, see arrow). The *in situ* expression pattern in the larval gut is mimicked by two Gal4 lines driving UAS-RedStinger (nuclear RFP, in red). *miple1*-Gal4 shows expression in a group of cells in the anterior part of the midgut (O, see arrow) but no expression in other parts of the gut (N), while *miple2*-Gal4 shows expression in AMPs in the larval midgut (P and Q, see arrow). Asterix (* in P) indicates ectopic Gal4 expression from observed in the proventriculus. Nuclear stain in (N-Q) visualises the gut (DAPI, white).

To investigate the functional roles of the *Drosophila* MDK/PTN homologues *miple1* and *miple2 in vivo*, we generated deletion mutants in the *miple1* and *miple2* loci, which are tandemly situated at 61B3 on the left arm of the third chromosome (Figure 1A). To do this we performed an imprecise excision screen of a P-element (*P{EPgy2}^EY04126^*) situated between *miple1* and *miple2* (Figure 1A). This approach led to the identification of single *miple1* (*miple1^Δ194^*) and *miple2* (*miple2^Δ396^*) null mutants (Figure1 B, C) that do not produce detectable RNA (data not shown). Examination of both *miple1^Δ194^ and miple2^Δ396^* mutants revealed no obvious gross abnormalities, and flies were healthy, fertile and viable as homozygous stocks.

Given that Miple1 and Miple2 proteins display rather distinct expression patterns during embryogenesis, it is reasonable to expect that these small secreted molecules may be functionally redundant. This has been observed previously in *Drosophila* for example in the case of the FGF-like proteins Thisbe and Pyramus whose overlapping role is only seen when both genes are inactivated [58]. In order to delete *miple1* and *miple2* simultaneously we performed the excision screen in mutants lacking the DNA helicase DmBlm (*Blm^D2^/Blm^D3^* background). This approach led to the isolation of *miple1,miple2^Δ104^* mutant animals with a complete deletion of both *miple1* and *miple2* genomic regions (Figure 1B). The *miple1,miple2^Δ104^* deletion also partially deletes the downstream neighboring gene *tiny tim 2 (ttm2),* a gene reported to be exclusively expressed in testis [59]. All generated deletion mutants of the *miple* genes are viable, although the *miple1,miple2^Δ104^* males are sterile due to loss of Ttm2 (see below). We further analysed the survival of progeny from females lacking the maternal contribution of *miple* mRNA by crossing homozygous mutant females to deficiency males, obtaining the expected Mendelian rations in both male and female progeny (Figure S1).

### Loss of *ttm2* but not *miple1* and *miple2* results in male sterility

To investigate a possible function of the *ttm2* gene and its contribution to the sterility phenotype of *miple1,miple2^Δ104^* animals we analysed progeny from crosses with either one of the two deficiency strains *Df(3L)BSC125* (that uncovers *miple1*, *miple2*, *ttm2* and 5 additional genes) and *Df(3L)BSC126* (which uncovers *miple2* and *ttm2* and 6 additional genes). Trans-heterozygous *Df(3L)BSC125/Df(3L)BSC126* males deficient in both *miple2* and *ttm2* are male sterile (Table 1, Table S1). Male sterility is also observed in *miple1,miple2^Δ104^* homozygous flies and cannot be rescued by ectopic over-expression of Miple2 (*miple2*-*Gal4>UAS-miple2*) in the *miple1,miple2^Δ104^* mutant background (Table 1, Table S1), suggesting that the *miple1,miple2^Δ104^* mutants lack functional Ttm2 protein. A putative *ttm2* allele, a P-element insertion *(PBac{3PHy+}C205)*, positioned downstream of *miple2* and upstream of *ttm2* (Figure 1A), was also examined. Homozygous *PBac{3PHy+}C205* males mated to virgin female *PBac{3PHy+}C205* or to control *w^1118^* or *control^rev657^* flies, were also sterile (Table S1). We further analysed this stock in allelic combinations with various *miple* alleles and deficiencies, concluding that this stock is male sterile and supporting a role for Ttm2 in male fertility in *Drosophila* in keeping with its previously described expression pattern. Analysis of the early fecundity of *miple1,miple2^Δ104^* females in comparison to control (*control^rev657^*) females suggests that loss of Miple proteins does not affect female fertility (Figure S2).

**Table 1:** Loss of *ttm2* but not *miple1* and *miple2* results in male sterility.

Allele combination	Genotype	Gene copy			Estimated fertile (mated to control*^rev657^* females)
		*miple1*	*miple2*	*ttm2*	
***miple*** ** alleles**	*miple1,miple2^Δ104^/Df(3L)BSC125*	*−/−*	*−/−*	*−/−*	Sterile
	*miple1,miple2^Δ104^/Df(3L)BSC126*	*+/−*	*−/−*	*−/−*	Sterile
	*Df(3L)BSC125/Df(3L)BSC126*	*+/−*	*−/−*	*−/−*	Sterile
***miple2*** ** rescue**	*miple2-Gal4; UAS-miple2; miple1,miple2^Δ104^/miple1,miple2^Δ104^*	*−/−*	*+/−*	*−/−*	Sterile
***ttm2*** ** alleles**	*PBac{3HPy^+^}C205/PBac{3HPy^+^}C205*	*+/+*	*+/+*	*−/−*	Sterile
	*miple1,miple2^Δ104^/PBac{3HPy^+^}C205*	*+/−*	*+/−*	*−/−*	Sterile
	*Df(3L)BSC125/PBac{3HPy^+^}C205*	*+/−*	*+/−*	*−/−*	Sterile
	*Df(3L)BSC126/PBac{3HPy^+^}C205*	*+/+*	*+/−*	*−/−*	Sterile

### Characterisation of *miple1* and *miple2* expression in larval tissues

The embryonic mRNA expression pattern of *miple1* and *miple2* mRNA has been described previously [27]. Briefly, miple1 RNA is mainly expressed in the CNS, while miple2 RNA is expressed in a broader pattern in both the developing endoderm and in the CNS in addition to the presence of a strong maternal contribution. We extended the analysis of *miple* mRNA expression to L3 larvae, where we detected strong expression of *miple1* RNA in the neuropile (data not shown) of early L3 larvae, and in the optic lobes of late L3 larvae (Figure 1D).

Weak *miple1* mRNA expression can also be detected in imaginal discs (Figure 1E and F) and in a distinct population of cells in the anterior gut (Figure 1K) although no detectable expression could be seen in any other region of the larval gut (Figure 1J). The *miple1* mRNA pattern is further supported by the activity of a *miple1-Gal4* transgenic driver that displays similar activity in the larval gut (Figure 1N and O, see arrow). Robust expression of *miple2* mRNA was detected in all L3 larval imaginal discs (Figure 1H and I), while the CNS (Figure 1G) does not show any detectable *miple2* expression. The early L3 larval gut shows discrete *miple2* RNA expression in a subset of cells (Figure 1L and M, see arrows), particularly in potential adult midgut progenitor cells (AMPs), a pattern supported by *miple2-Gal4* driven RedStinger (nRFP) which is expressed in only smaller but not in large nuclei (Figure 1P and Q, see arrows). The identity of these cells as AMPs was further confirmed with the AMP marker *esg-lacZ* (data not shown). These results suggest that both genes are strongly expressed after embryonic stages and present a combined pattern of broad expression in L3 larval tissues.

### Miple1 and Miple2 are secreted heparin binding proteins

The residues identified in human MDK and PTN required for these proteins to bind heparin are not fully conserved in the *Drosophila* Miple1 and Miple2 [25]. However both Miple proteins are enriched in basic amino acids such as arginine and lysine, which suggests that the *Drosophila* Miple proteins may also bind heparin and subsequently have the capacity to bind heparin sulphate proteoglycan (HSPG) proteins. We employed HEK293 cells to express secreted Miple1 and Miple2 and examined binding to heparin-agarose *in vitro*, using human MDK and PTN as positive controls (Figure S3). These results show that both *Drosophila* Miple1 and Miple2 proteins exhibit heparin binding properties *in vitro* and may therefore share a conserved HSPG binding ability previously described for human MDK and PTN.

### Neither Miple1 nor Miple2 are critical for the activation of Alk signaling in the developing visceral mesoderm

The genetically tractable *Drosophila* model offers an excellent opportunity to explore the suggested role for the *miple* homologues PTN and MDK as ligands for the ALK receptor tyrosine kinase in vertebrates. We performed a detailed analysis of the embryonic visceral mesoderm in single and double *miple* mutant embryos, including activation of MAPK signaling (dpERK) mediated by Alk signaling in FCs [27]–[29], the founder cell specific marker Org-1 [52] and the fusion competent myoblast specific factor Vrp1 [53].

**Figure 2:**
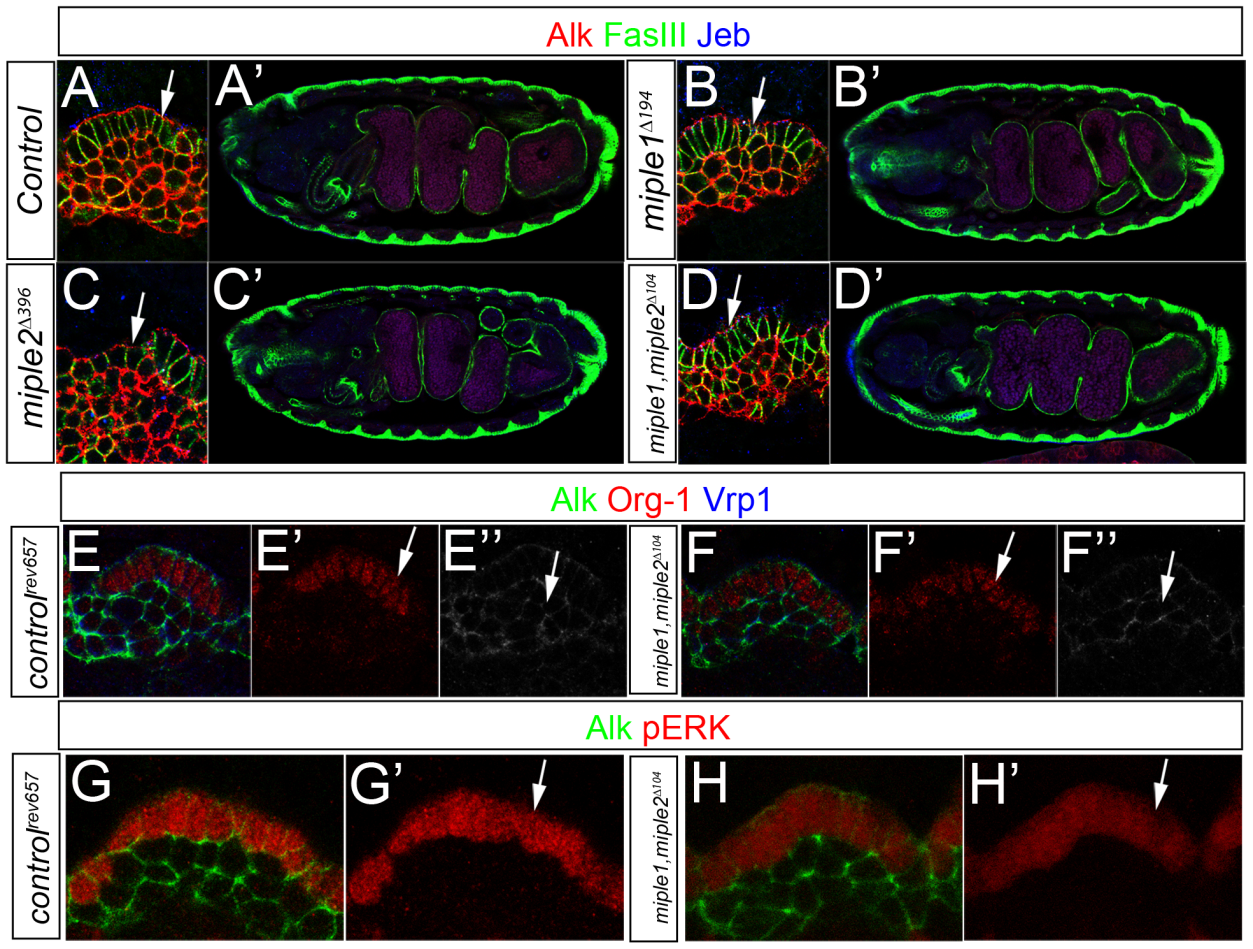
Miple proteins are not required for Alk signaling in the embryonic visceral mesoderm. (**A-D**) The deletion mutants *miple1^Δ194^*, *miple2^Δ396^* and *miple1,miple2^Δ104^* exhibit normal visceral mesoderm development. In *miple1^Δ194^*, *miple2^Δ396^* and *miple1,miple2^Δ104^* deletion mutants, Alk positive columnar shaped founder cells (FCs, arrows) and fusion competent myoblasts (FCMs) are correctly specified (A, B, C, and D). At embryonic stage 17 midgut chambers are normally formed in all three mutants (B′, C′ and D′) and are comparable to control embryos (A′). Embryos are stained with Alk (in red), Jeb (in blue) and FasIII (in green). (**E-F**) At late stage 10 double *miple1,miple2^Δ104^* deletion mutant embryos express FC (F′, Org-1 in red, arrow) and FCM (F′′, Vrp1 in white, arrow) specific markers as *control^rev657^* (E′, E′′). (**G-H**) At late stage 10, *miple1,miple2^Δ104^* double mutant embryos display wild type ERK activity in FCs (H′, pERK in red, arrow) comparable with *control^rev657^* revertant control FCs (G, G′). Embryos are stained with Alk (in green) and Org-1 or pERK as indicated (in red).

To remove the maternal and zygotic contributions of both *miple1* and *miple2*, double mutant *miple1,miple2^Δ104^*, homozygous females were crossed with *Df(3L)BSC125* males which harbor a deficiency removing the entire *miple1* and *miple2* loci. Examination of visceral mesoderm morphology in *miple1,miple2^Δ104^/Df(3L)BSC125* was performed in both, single *miple1* and *miple2* mutants as well as in double *miple1,miple2* mutants. In early stage embryos we clearly detect the layer of columnar FCs and the more round FCMs in the developing VM indicating proper specification of the two cells types in both mutant and control embryos (Figure 2, compare B, C and D with control A, arrows indicate FCs). Similarly, in late stage embryos we detect no obvious abnormalities in gut formation, as the four midgut chambers are properly formed and organised in embryos of all mutants analysed (Figure 2, compare B′, C′ and D′ with control in A′).

The specification of visceral FCs is dependent on Alk signaling and one protein that is expressed in a restricted pattern in FCs is the transcription factor Org-1 [28], [52]. We analysed the Org-1 expression as well as the expression of Verprolin 1 (Vrp-1/WIP/Sltr) [53], [60], [61], a co-regulator of the Arp2/3 complex that is expressed exclusively in FCMs, to analyse if cell type determination is affected in *miple* mutants. Org-1 expression is restricted to FCs in both double mutant and control embryos (Figure 2, compare F′ with control in E′, see arrow), while Vrp-1 is exclusively observed in FCMs (Figure 2, compare E′′ with control in F′′, see arrow). Activation of Alk leads to MAPK activation which is visualised by detection of phosphorylation of MAPK(ERK) (pERK) in FCs. We observed robust MAPK activation in FCs of *miple1,miple2^Δ104^* mutant embryos (Figure 2, compare H′ with control in G′, see arrow). Thus, we conclude that Miple proteins are not required for Alk signaling during embryogenesis *in vivo* as measured by founder cell specification in the developing *Drosophila* visceral mesoderm.

### Over-expression of Miple proteins is not sufficient to drive Alk activation *in vivo* in the fruitfly

**Figure 3:**
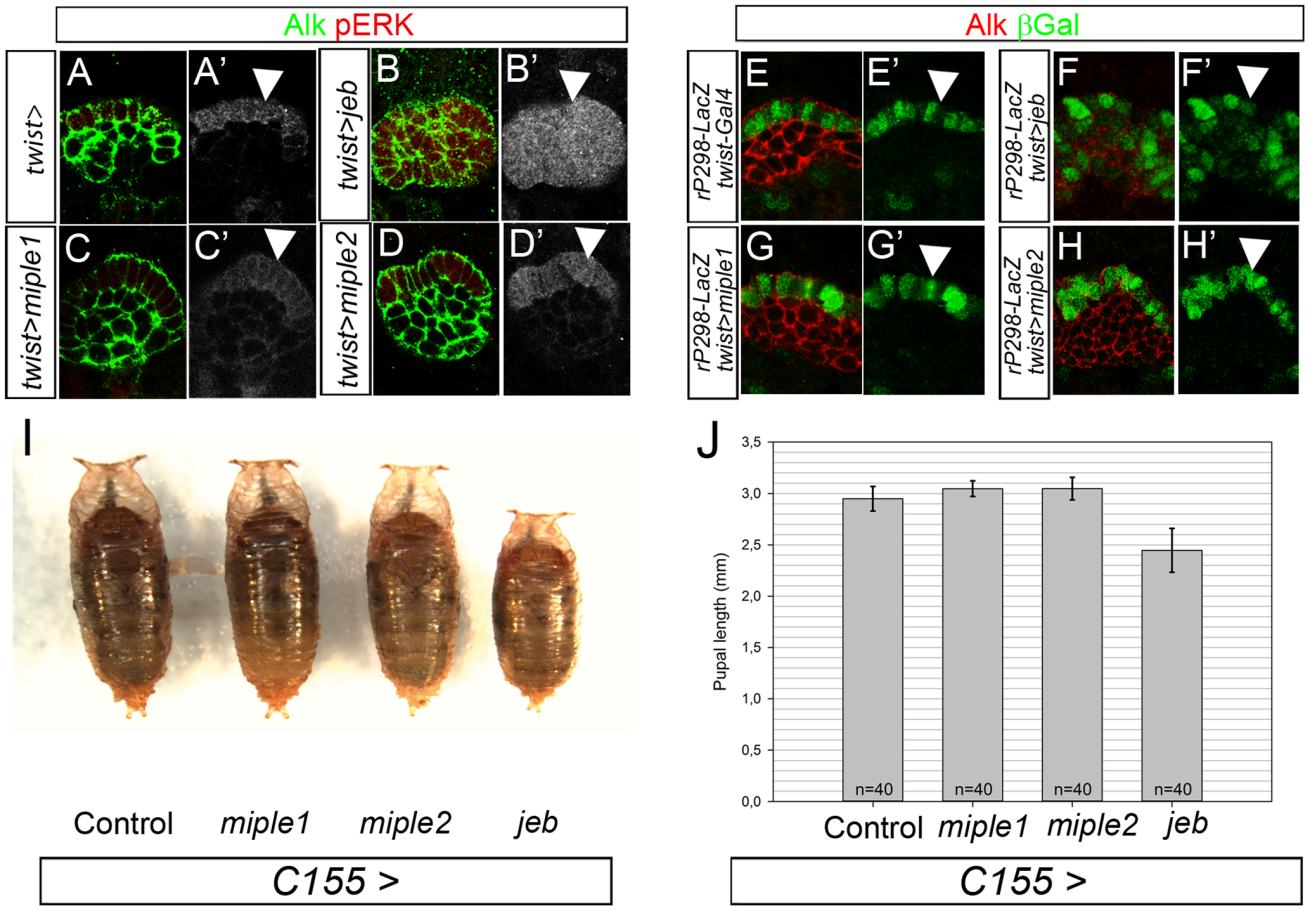
Ectopic expression of Miple proteins does not activate Alk signaling. (**A-D**) Ectopic expression of Miple1 (C, C′) or Miple2 (D, D′) with *twist2xPE-Gal4* in stage 10 embryos fails to ectopically activate ERK (pERK) in Alk positive visceral mesoderm. This is in contrast to ectopic expression of Jeb which is sufficient to activate ERK (pERK) in all Alk positive cells of the visceral mesoderm (B′ compare with C′ and D′, arrowhead). (**E-H**) Ectopic expression of Miple1 (G, G′) or Miple2 (H, H′) with *twist2xPE-Gal4* fails to ectopically activate the *duf/kirre* enhancer trap *rP298-LacZ*. As observed with pERK above (B′), Jeb is sufficient to activate robust LacZ reporter expression in all Alk positive cells of the visceral mesoderm (F′ compare with G′ and H′, arrowhead). (**I-J**) Ectopic expression of Miple proteins does not affect pupal size during development. Expression of Jeb with the pan-neuronal driver (*C155-Gal4*) results in a reduction of pupal length (mm) in comparison to controls. In contrast, pan-neuronal expression of Miple1 or Miple2 does not affect pupal size. Representative pupae are shown in (I). Quantification is shown in (J), error bars denote S.E.M. (n = 40). All pupae analysed were female, confirmed by analysis of hatched adults.

Our results show that endogenous Alk signaling in the developing visceral mesoderm is not impaired by loss of Miple proteins *in vivo*. Next, we examined whether over-expression of Miple proteins with the Gal4/UAS system [62] is sufficient to drive ectopic Alk activation. This can be tested since all cells of the developing visceral mesoderm express Alk, while only those cells exposed to the Jeb ligand activate robust Alk signaling. Thus ectopic over-expression of the Alk ligand Jeb using the *twist2xPE-Gal4* driver leads to ectopic activation of MAPK in all cells of the visceral mesoderm (Figure 3B) [28] in contrast to the normal pERK pattern which is restricted to the FC population (Figure 3A). We next asked whether activation of MAPK could be expanded to all VM cells by ectopic expression of either Miple1 or Miple2. We did not observe any ectoptic activation of MAPK upon over-expression of Miple proteins in the visceral mesoderm (Figure 3C and D). Consistent with the results above, over-expression of Jeb using the *twist2xPE-Gal4* leads to robust activation of the FC-specific *rP298-lacZ* enhancer trap [46] in all Alk positive cells of the VM (Figure 3F). In contrast, over-expression of Miple1 and Miple2 does not affect *rP298-lacZ* expression (Figure 3G and H), which is comparable with the restricted founder cell expression observed in controls (Figure 3E).

To analyse potential Alk activation by Miple proteins in a different tissue context we investigated the effects of Alk signaling on cell growth. Alk and Jeb have been reported to regulate body size determination [39], [40], where over-expression of the ligand Jeb (as well as over-expression of wild type Alk and constitutively active Alk) in pan neuronal tissues using *C155-Gal4* dramatically decreases body size. We therefore analysed the pupal size of *miple* mutants in comparison to controls. The results clearly show that loss of Miple proteins does not affect body size determination, at the level of pupal length (Figure S4). We next asked whether ectopic expression of Miple proteins effect body size determination by ectopically expressing Miple proteins in pan-neuronal tissues. In contrast to the effect seen on Jeb expression, we did not observe any effect on size, suggesting that Miple proteins are unable to activate Alk in this cellular context (Figure 3I, quantified in J).

**Figure 4:**
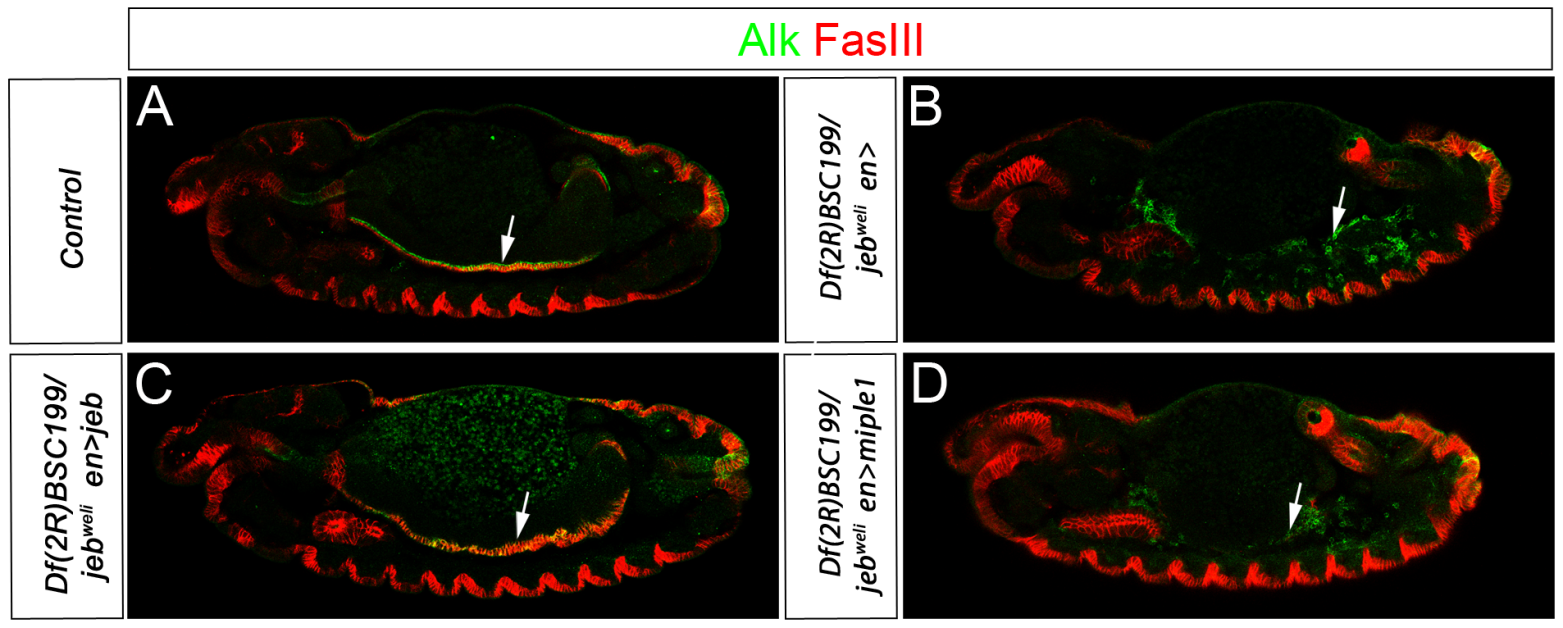
Miple proteins do not recues the loss of Jeb phenotype in the visceral mesoderm. (**A-D**) In *jeb* mutant embryos (*Df(3L)BSC199/jeb^weli^*) VM founder cells (green) are not specified. Subsequently, the VM fails to undergo fusion (arrow in B; compare with control in A where Alk and FasIII (red) positive VM is well developed). Ectopic expression of Jeb in *jeb* mutant embryos rescues the VM developmental defect (arrow in C, compare with A and B), while expression of Miple1 is unable to rescue the *jeb* mutant phenotype (arrow in D).

In a final attempt to examine the hypothesis that Miple proteins may drive Alk activation we tried to rescue the *jeb* mutant embryonic gut phenotype with Miple over-expression. To do this either Miple1 (Figure 4D) or Miple2 (not shown) were expressed in a *jeb* mutant background using the *en-Gal4* (*engrailed*-*Gal4*) driver. Clearly, even elevated levels of Miple1 (Figure 4D) or Miple2 (not shown) are completely unable to activate Alk and rescue the formation of the visceral mesoderm in absence of Jeb protein (Figure 4D). Control animals overexpressing Jeb protein allowed rescue of the visceral muscle structure at stage 14 (Figure 4C), and resulting in a portion of animals (19,5%, n = 340) that developed into adult flies (Figure S5).

Taken together, we find no *in vivo* evidence for the activation of Alk signaling in developing VM by Miple proteins. First, flies lacking both Miple1 and Miple2 are viable and fertile with no apparent defects in Alk signaling in the developing visceral mesoderm. Second, over-expression of Miple proteins is unable to drive Alk signaling in two independent processes. Third, excessive levels of Miple proteins are unable to activate Alk and rescue the *jeb* mutant gut phenotype *in vivo.* Thus, our data suggest that neither of these small heparin-binding proteins function as Alk activating ligands in the *Drosophila* tissues we have analysed *in vivo*.

### Miple1 and Miple2 affect adult lifespan and Miple1 is expressed in the adult brain

**Figure 5:**
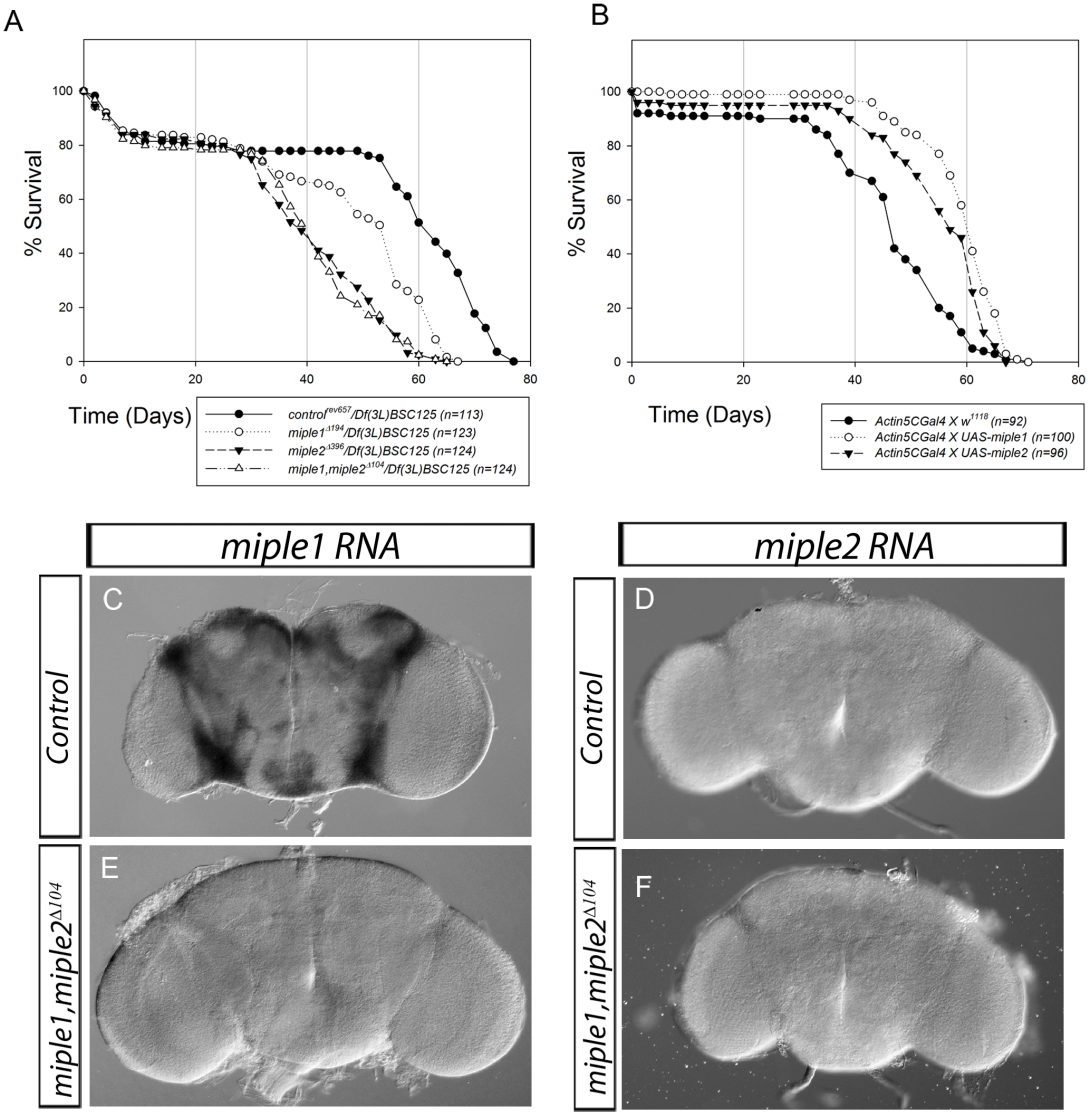
Gain and loss of Miple expression affect lifespan in *Drosophila*. (**A**) Loss of Miple proteins affect lifespan. Decreased mean lifespan is observed in *miple1^Δ194^/Df(3L)BSC125* (open circles) and *miple2^Δ396^/Df(3L)BSC125* (filled triangles) single mutant flies as well as *miple1,miple2^Δ104^/Df(3L)BSC125* (open triangles) double mutants. Revertant *control^rev^* ^657^ */Df(3L)BSC125* (filled circles) were employed as control (**B**) Ectopic over-expression of both Miple1 (open circles) and Miple2 (filled triangles) with *actin5C-Gal4* increases mean lifespan in comparison to controls (filled circles). All analysed flies were females. Number of flies is indicated as n. (**C-F**) *miple1* mRNA is strongly expressed in adult brain. No expression of *miple1* mRNA is detected in *miple1,miple2^Δ104^* double mutant brains (E). *miple2* mRNA *in situ* in adult brains. No *miple2* mRNA expression is detected in either wildtype (D) or *miple1,miple2^Δ104^* double mutant control brains (F).

While *miple* mutant flies are viable they have a decreased lifespan, as observed in both single *miple2^Δ396^* and *miple1, miple2^Δ104^* mutants when compared with *control^rev657^* (Figure 5A). An intermediate effect on mean lifespan was seen in *miple1^Δ194^* mutants (Figure 5A). This observation led us to examine whether over-expression of Miple proteins may have an opposite effect, i.e. increased lifespan. Examining the effect of Miple expression under the control of a range of *Gal4* drivers we did not score any gross visible phenotypes (Table S2), suggesting that elevated levels of Miple proteins are well tolerated in the fly. However, we did observe a clear prolongation effect on lifespan in animals expressing Miple proteins with *actin5C-Gal4* (Figure 5B), and the pan-neuronal *C155-Gal4* driver (data not shown), indicating a novel function for these two proteins in the regulation of lifespan *in vivo*. While both proteins may exert similar functional effects on lifespan, the differences in their expression patterns may underlie the difference between loss of Miple1 and Miple2 animals in terms of lifespan. The observed effect on lifespan suggests that Miple1 and Miple2 have a function in adults and thus may be expressed during adult stages. Distinct expression of *miple1* mRNA was detected in adult brains (Figure 5C). This staining is absent in *miple1, miple2^Δ104^* double mutants (Figure 5E). In contrast to the robust *miple1* expression we were unable to *miple2* mRNA expression in adult brain (Figure 5D).

### Ectopic expression of Miple1 or Miple2 in the eye does not affect ommatidia organisation

We next analysed the eye morphology of the different *miple* mutants and observed that the ommatidia structure in flies lacking either one of the *miple* genes or both is normal (Figure S6, compare B, C and D with control in A). Additionally, we analysed the effect of over-expression of multiple independent *UAS-miple1* and *UAS-miple2* transgenic lines in the developing eye with *GMR-Gal4*, and did not observe effects on adult eye morphology with either Miple1 or Miple2 in comparison to the control (Figure S6, compare F, G and H with control in E). In each case Miple protein expression was confirmed by either immunoflouresence or immunoblotting (Figure S7). Our results here are in contrast to those of [63] that detected a clear effect on eye morphology upon over-expression of Miple1 in the eye. We do not observe such an affect despite clear expression of Miple proteins from our transgenes. It is possible that the differences in results observed here are a result of differences in expression levels, or due to other currently unclear reasons.

**Figure 6:**
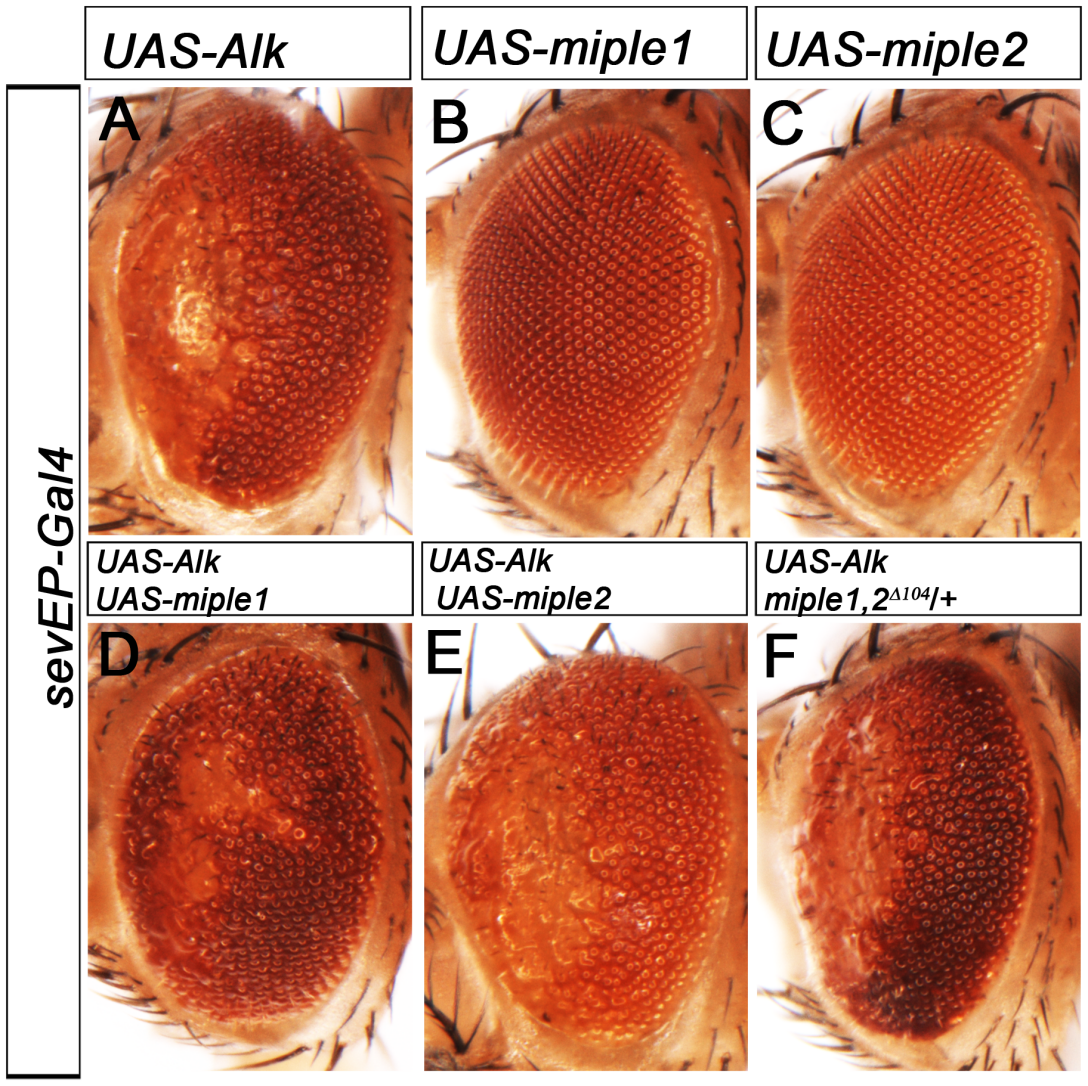
Ectopic co-expression of Miple1 and Miple2 in the developing eye does not modify phenotypes caused by enhanced Alk activity. (A-F) Ectopic expression of *Drosophila* Alk with *sevEP-Gal4* disrupts the eye morphology and leads to loss of ommatidia in the anterior region of the eye (A). Expression of Miple1 (B) or Miple2 (C) alone does not disrupt eye morphology. Ectopic expression of Alk together with either Miple1 or Miple2 protein in the developing eye neither enhances nor suppresses the eye phenotype caused by ectopic Alk expression (D, E). Removing one copy of both *miple* loci with the *miple1,miple2^Δ104^* deficiency does not modify the *sevEP-Gal4>UAS-Alk* phenotype (F).

To test whether MDK/PTN/Miple protein expression together with Alk could modify either *Drosophila* or human Alk in the *Drosophila* eye, we ectopically expressed Miple proteins together with Alk, using the *sevEP-Gal4* driver. Expression of Alk alone causes a characteristic eye phenotype with loss of defined ommatidia in the anterior region of the eye (Figure 6A). Expression of the two Miple proteins alone, as described above with *GMR-Gal4*, does not disrupt eye morphology (Figure S6F, G), and no enhancement or suppression of the *sevEP-Gal4>UAS-Alk* phenotype was observed upon co-expression with either Miple1 or Miple2 (Figure 6D, E compare with control 6A). Furthermore, the *sevEP-Gal4>UAS-Alk* induced eye phenotype is not modified by reduction of *miple1* and *miple2* (Figure 6F).

**Figure 7:**
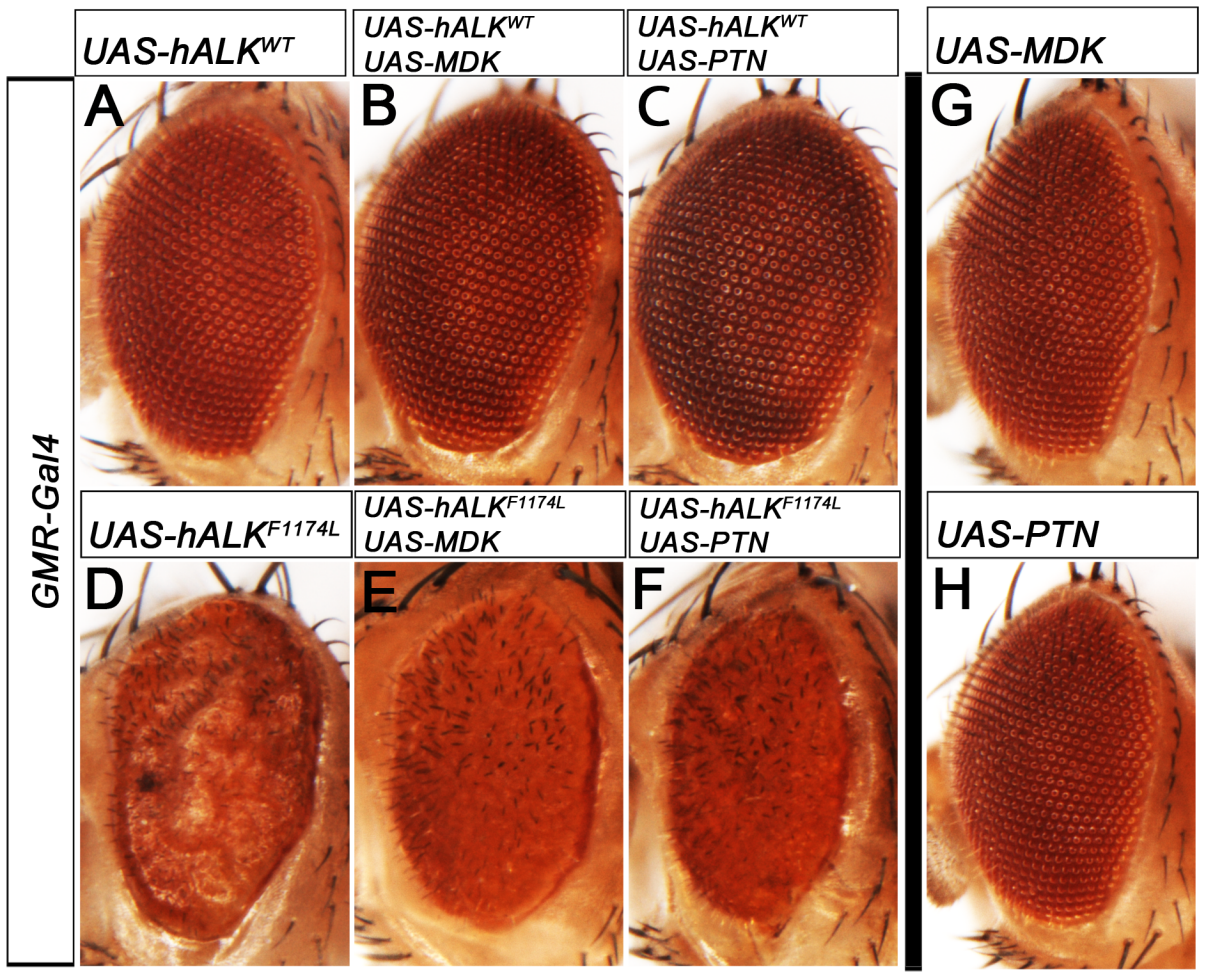
Combined expression of the human Miple homologues (MDK and PTN) and human ALK does not affect eye morphology in *Drosophila*. (A-H) Ectopic expression of human wildtype ALK (A) in developing eye using *GMR-Gal4* does not affect eye morphology as no functional ligand is present in the fly. Ectopic expression of human MDK and PTN in the developing eye, alone (G, H) and in combination with wildtype human ALK (B, C), using *GMR-Gal4* does not affect adult eye morphology. Neither MDK (E) nor PTN (F) modify the eye phenotype caused by expressing a constitutively active human ALK (hALK F1174L gain-of-function mutation observed in human neuroblastoma) (D).

To further address if this family of ligands can activate the Alk receptor *in vivo*, we extended these experiments to include human MDK and PTN employing both *GMR-Gal4* (Figure 7) and *sevEP-Gal4* (Figure S8) together with human ALK. Over-expression of wild type hALK alone does not affect eye morphology (Figure 7A) [64], as human ALK does not recognise the *Drosophila* Alk ligand, Jeb [65]. This is in contrast to the expression of a constitutively active gain-of-function hALK^F1174L^ mutant that strongly disrupts eye morphology (Figure 7D) [64]. We could not detect any visible effect on eye morphology upon expression of MDK or PTN together with wild type hALK (Figure 7B and C) suggesting that the receptor is not activated in this context. Further, when expressing MDK and PTN with the gain of function hALK^F1174L^ allele (Figure 7E and F) we did not observe a modified eye phenotype. Protein expression by MDK and PTN transgenes was confirmed by immunofluoresence (Figure S7).

Together these results suggest that neither the *Drosophila* Alk nor the human ALK receptor are directly activated *in vivo* by the human growth factors MDK/PTN or the *Drosophila* counterparts Miple1 and Miple2.

## Discussion

In this work we have characterised the *Drosophila* homologues of the MDK/PTN family of small heparin binding proteins – Miple1 and Miple2, by generating deletion mutants of both *miple1* and *miple2* and a larger deletion encompassing both genes. Removal of both *miple* genes does not affect viability, thus these genes appear to perform non-essential functions and are dispensible for normal development of the fruitfly. Similarly to MDK and PTN, we observe that Miple1 and Miple2 are secreted proteins that display heparin binding *in vitro*. Thus, a receptor for Miple proteins in the fly potentially dimerises with or functions in a receptor complex with proteins containing Heparin Sulphate Proteoglycan (HSPG) motifs. In fact, multiple receptors for the MDK/PTN family of cytokines have been reported, including the Receptor-like Protein Tyrosine Phosphatase ζ^∼^β (RPTPζ/RPTPβ [66], N-syndecan [67], [68], Low-Density-Lipoprotein (LDL) Receptor-related Protein 1 (LRP1) [69], Anaplastic Lymphoma Kinase (ALK) [14]–[16] the α_4_β_1_- and α_6_β_1_-integrins [70] and membrane localised Nucleolin [71] with no clear consensus concerning receptors responding to ligand activation by MDK or PTN *in vivo*.

Reports concerning ALK as a candidate receptor for MDK/PTN are contradictory [12], with some supporting MDK and PTN as functional ligands [14]–[17], as well as articles reporting no interaction [18], [19]. A single *Drosophila* homologue of ALK exists [44], permitting *in vivo* analysis of this receptor tyrosine kinase. Here, we have addressed the question of whether *Drosophila* MDK/PTN homologues Miple1 and Miple2 are functional ligands for Alk. Analysis of *miple* mutants suggest that these factors do not play an important function in Alk signaling dependant processes such as specification of founder cells in the visceral mesoderm during embryogenesis in the fruitfly. The effect of *miple* on body size regulation, a process regulated by Alk signaling [39], was also examined in this work. In contrast to manipulation of the levels of the ALK ligand Jeb, neither loss of *miple* gene expression nor ectopic expression of Miple proteins affected the regulation of body size in flies. It is possible that a role for the Miple proteins exists in other Alk related processes such as photoreceptor axonal targeting [35], neuronal transmission [37], brain sparing [38], ethanol sensitivity [41], memory and learning [39], [40] but these have not been examined in this study.

One phenotype observed in our mutants was that of male sterility, however further investigation revealed this to be due to the loss of the *ttm2* locus, and was confirmed by genetic analysis. Interestingly, our findings that *miple* double mutant females are fertile indicates that loss of Miple proteins do not effect female fertility. This is in contrast to the infertility phenotype previously reported in female PTN/MK double knockout mice [72].

While no gross phenotypes are apparent in *miple* double mutant animals, we observed a reduced mean lifespan in *miple2* single mutants as well in *miple* double mutants, suggesting a role for Miples in regulation of lifespan. This function is further strengthened by the increase in mean lifespan resulting from ectopic over-expression of Miple1 or Miple2. Robust *miple1* mRNA expression was detected in brains of adult flies, while *miple2* was not detected in brains. This is in agreement with the expression observed in larval tissues. The strong *miple1* mRNA expression suggests a role for Miple1 in re- or degeneration of neurons, something that has been reported for both PTN [73] [74] [75] and MDK [76] [77].

The increase in lifespan may function on a number of levels, with one other hypothesis based on potentially altered immune function. This is suggested by recent studies reporting bactericidal [78] and fungicidal [79] activities of MDK and PTN *in vitro,* that are conserved for Miple2 *in vitro* [78]. Thus increased levels of Miple proteins would not necessarily be harmful for the fly, but may exert a positive effect. Whether Miple1 and Miple2 perform such functions *in vivo* in the fly are currently unknown and the mechanisms underlying their effect on lifespan require further more detailed investigation. When considering other receptors proposed for MDK/PTN, and whether their fly homologues potentially function as receptors for Miple1 and Miple2, it is interesting to note that hypomorphic syndecan (*sdc)* alleles have been reported to have a reduced lifespan and display a number of metabolic phenotypes [55]. Further, alleles of the βPS integrin subunit, *myospheroid,* show the opposite effect with increased life span in heterozygous condition [80]. Like the *miple* mutants, the closest fly homologue of the *rptpβ/ζ* receptor tyrosine phosphatase receptor, encoded by *ptp99A*, is homozygous viable [81]. Viable mutants have also been reported for *lrp-1*, although no further characterisation has been described [82]. In this study, we have not experimentally addressed whether any of these candidates are functional ligands for Miple1 and Miple2, indeed future studies should address these possibilities.

Our findings in this study do not support previous reports that MDK and PTN directly activate ALK, but are in agreement with other studies that show that MDK and PTN do not activate ALK. Precisely what functional roles Miple1 and Miple2 play in the fly remains unclear, but we can conclude that they are not of critical developmental importance, despite their extensive expression during development. Future work will be required to address their *in vivo* targets and function.

## Supporting Information

Figure S1 **Deletion of** ***miple1*** **and** ***miple2*** **does not affect survival of maternal zygotic mutant flies.** Graph showing the average percentage of progeny, with homozygous mutant (over *Df(3L)BSC125*, black bar) and heterozygous mutant (over *TM3 actinGFP* balancer, grey bar) genotype and for female (black bar) and male (grey bar) of the adult progeny, from crosses between homozygous mutant females and heterozygous *Df(3L)BSC125/TM3 actinGFP* males. The ratio between homozygous/heterozygous and female/male adult progeny is comparable to *miple^657rev^* control and is the expected mendelian ratio for both single *miple* mutants and double *miple* mutants. The average percentage of progeny of the different genotypes and sex, was calculated from 3 biological replicates for each genotype with total number flies (n) from all replicates indicated.
(TIF)

Figure S2 **Loss of Miple does not affect early female fecundity as measured by embryo production.** Graph indicating female fecundity as measured by number of eggs laid per female per day (24 hours) over 5 days. Homozygous *miple1, miple2^Δ104^* double mutant flies (two replicates, light and dark grey bars) were comparable to *control^rev^* ^657^ (black bar). Overall fecundity was observed to decrease over 5 days in both mutant and control flies.
(TIF)

Figure S3 **Miple proteins bind heparin** ***in vitro*** **.** Western blot analysis of Miple proteins bound to heparin-agarose. Miple1 protein was detected in the conditioned media fraction (20 µl) (input, *) as well as in the heparin-agarose eluted fraction (20 µl) (bound, arrowhead). Similar results were observed with Miple2-HA detected with either HA antibody or Miple2 antibody, although in the case of using anti-Miple2, a smaller, potentially degraded or processed Miple2 protein was detected (input, * and bound, arrowhead), while with anti-HA several smaller bands was detected, suggesting a degradation of bound Miple2 protein. The human MDK and human PTN proteins are clearly detected in conditioned media (input,*) as well as in the heparin eluate (bound, arrowhead).
(TIF)

Figure S4 **Loss of Miple does not affect pupal size.** Progeny from maternal zygotic *miple* mutant females crossed to males bearing the deficiency *Df(3L)BSC125*, were measured at late pupal stages. Pupae length (in mm) of *miple1^Δ194^/Df(3L)BSC125* and *miple2^Δ396^/Df(3L)BSC125* single mutants as well as double deficient *miple1,miple2^Δ104^/Df(3L)BSC125* were comparable to control (*control^rev657^/Df(3L)BSC125*). All analysed pupae were confirmed as female and error bars denote S.E.M.
(TIF)

Figure S5 **Quantification of** ***jeb*** **mutant rescue experiments.** Quantification of *jeb* mutant rescue shows that ectopic expression of Jeb results in rescue to viable adult flies (19.5%, n = 340), while Miple1 and Miple2 both fail to rescue flies to adulthood. Percentage of rescue is calculated as 1/3 of progeny from the 3 genotypes that would be predicted to be homozygous viable per cross. Total number of flies analysed is denoted as n.
(TIF)

Figure S6 **Neither loss or gain Miple proteins result in obvious developmental phenotypes. (A-H)** Loss of Miple proteins or their over-expression, does not result in gross developmental defects, exemplified here by scanning EM of the *Drosophila* eye. No defects in ommatidal organization are observed in *miple1^Δ194^/Df(3L)BSC125* (B), *miple2^Δ396^/Df(3L)BSC125* (C) single mutant flies, or *miple1,miple2^Δ104^/Df(3L)BSC125* (D) double mutant flies. Revertant *control^rev^* ^657^ */Df(3L)BSC125* were employed as control (A). **(E-H)** Scanning EM photographs of adult eyes overexpressing *GMR-Gal4* driven Miple1 protein employing two independent *UAS-miple1* transgenic lines (F, G) and *UAS-miple2* (H). No effect on eye morphology was observed when compared with controls expressing GFP (E).
(TIF)

Figure S7 **Confirmation of expression of Miple1 and Miple2 as well as human MDK and PTN transgenes.** **(A)** To confirm expression of *UAS-miple1* transgenes, total protein from heads of newly hatched adult flies overexpressing Miple1 by the *GMR-Gal4* was analysed by western blotting. Miple1 can be detected for two independent transgene insertions but not in the negative control, showing that overexpressed and not endogenous Miple1 protein is detected. **(B)** Expression of Miple2.YFP.venus protein driven by *GMR-Gal*4 can clearly be detected in the larval eye disc (**C**) Expression of human PTN and human MDK protein in larval eye discs expressed by *GMR-Gal4* was confirmed by immunohistochemistry and show that these transgenes are functional.
(TIF)

Figure S8 ***sevEP-Gal4*** **driven expression of human Miple homologues MDK and PTN with hALK does not affect eye morphology.** **(A-F)** Ectopic expression of human PTN (C) and MDK (E) alone and co-expression with wild-type human ALK (D, F) in the in developing eye using *sevEP-Gal4* does not affect adult eye morphology. As positive control expression of constitutively active hALK F1174L gain-of-function mutation (observed in human neuroblastoma) was employed (B).
(TIF)

Table S1 **Fertility test of different allelic combinations of** ***miple*** **and** ***ttm2*** **mutant strains.** Males with the indicated allelic combinations were generated and crossed to female *control^rev^* ^657^ and scored for fertility measured by the presence of F1 1^st^ Instar larvae. Expression of *miple2* employing *miple2Gal4>UAS-miple2* in *miple1,mipleψ^Δ∼iψ^* mutant background fails to rescue the male sterile phenotype. All allelic combinations that produce sterile males contain homozygous deletion of the *ttm2* gene. Additionally a P-element insertion *PBac{3PHy+}C205* upstream of *ttm2* generates heterozygous fertile but homozygous sterile males and produces male sterility in transheterozygous combination with the three strains that carry deletions covering *ttm2* (*miple1,mipleψ^Δ∼iψ^*, Df(3L)BSC125 and Df(3L)BSC126).
(TIF)

Table S2 **Ectopic over-expression small screen of Miple transgenes.** A panel of various Gal4 strains was crossed to several independent UAS-*miple* transgenes at two different temperatures (25 and 29 degrees). The progeny from the indicated crosses was scored for phenotype after hatching. All tested transgenes in combination with all tested Gal4 reveal no obvious phenotypes, in table scored as NVP (No Visible Phenotype) indicating that over-expression of these transgenes is not harmful for the fly.
(TIF)
