# Supplementary figures and images for: The Drosophila Midkine/Pleiotrophin Homologues Miple1 and Miple2 Affect Adult Lifespan but Are Dispensable for Alk Signaling during Embryonic Gut Formation
Source: PLoS One. 2014 Nov 7;9(11):e112250. doi: 10.1371/journal.pone.0112250 (PMC4224452; doi:10.1371/journal.pone.0112250)

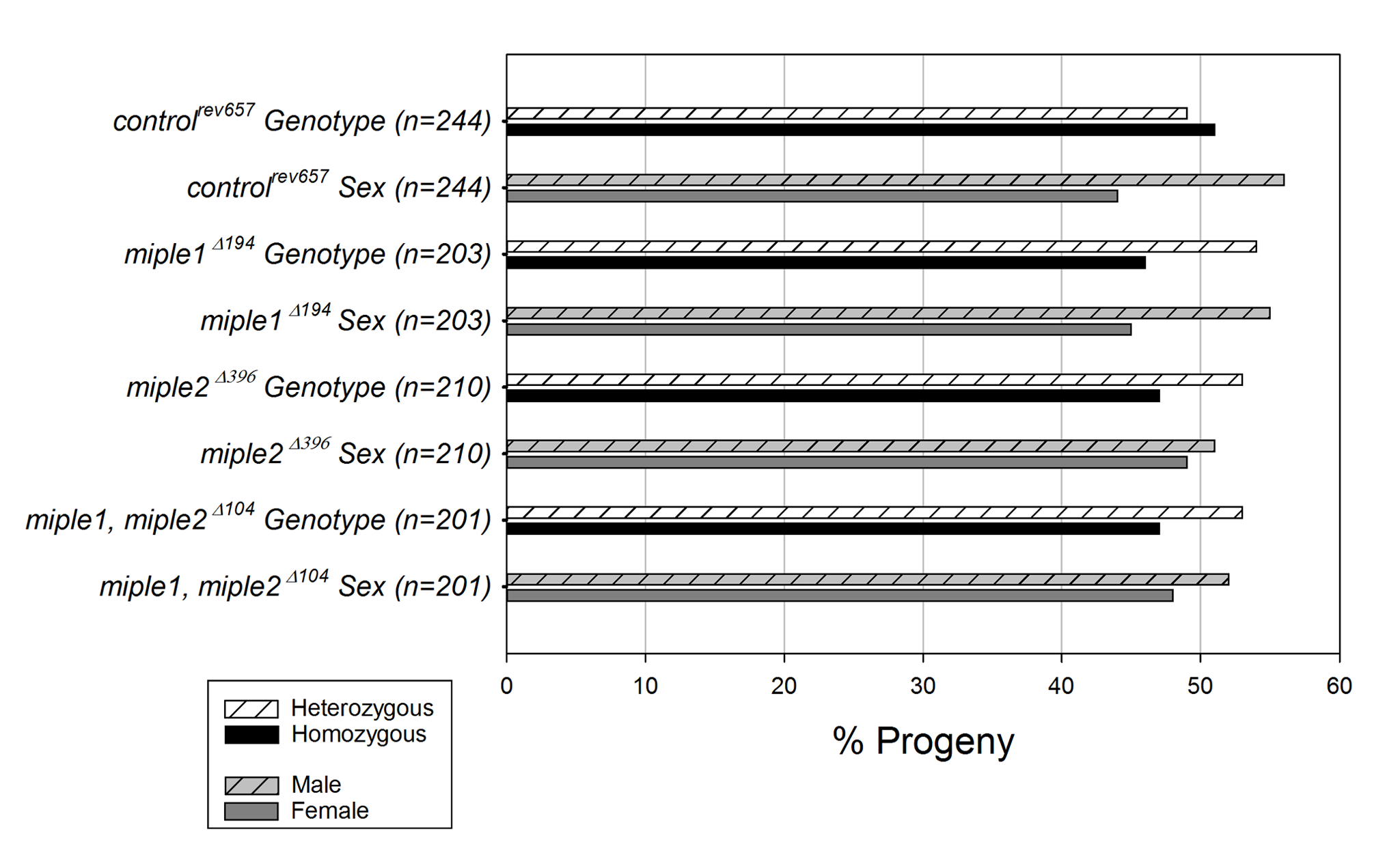

Supplement: Figure S1 — Deletion of miple1 and miple2 does not affect survival of maternal zygotic mutant flies. Graph showing the average percentage of progeny, with homozygous mutant (over Df(3L)BSC125, black bar) and heterozygous mutant (over TM3 actinGFP balancer, grey bar) genotype and for female (black bar) and male (grey bar) of the adult progeny, from crosses between homozygous mutant females and heterozygous Df(3L)BSC125/TM3 actinGFP males. The ratio between homozygous/heterozygous and female/male adult progeny is comparable to miple657rev control and is the expected mendelian ratio for both single miple mutants and double miple mutants. The average percentage of progeny of the different genotypes and sex, was calculated from 3 biological replicates for each genotype with total number flies (n) from all replicates indicated. (TIF) [file pone.0112250.s001.tif]

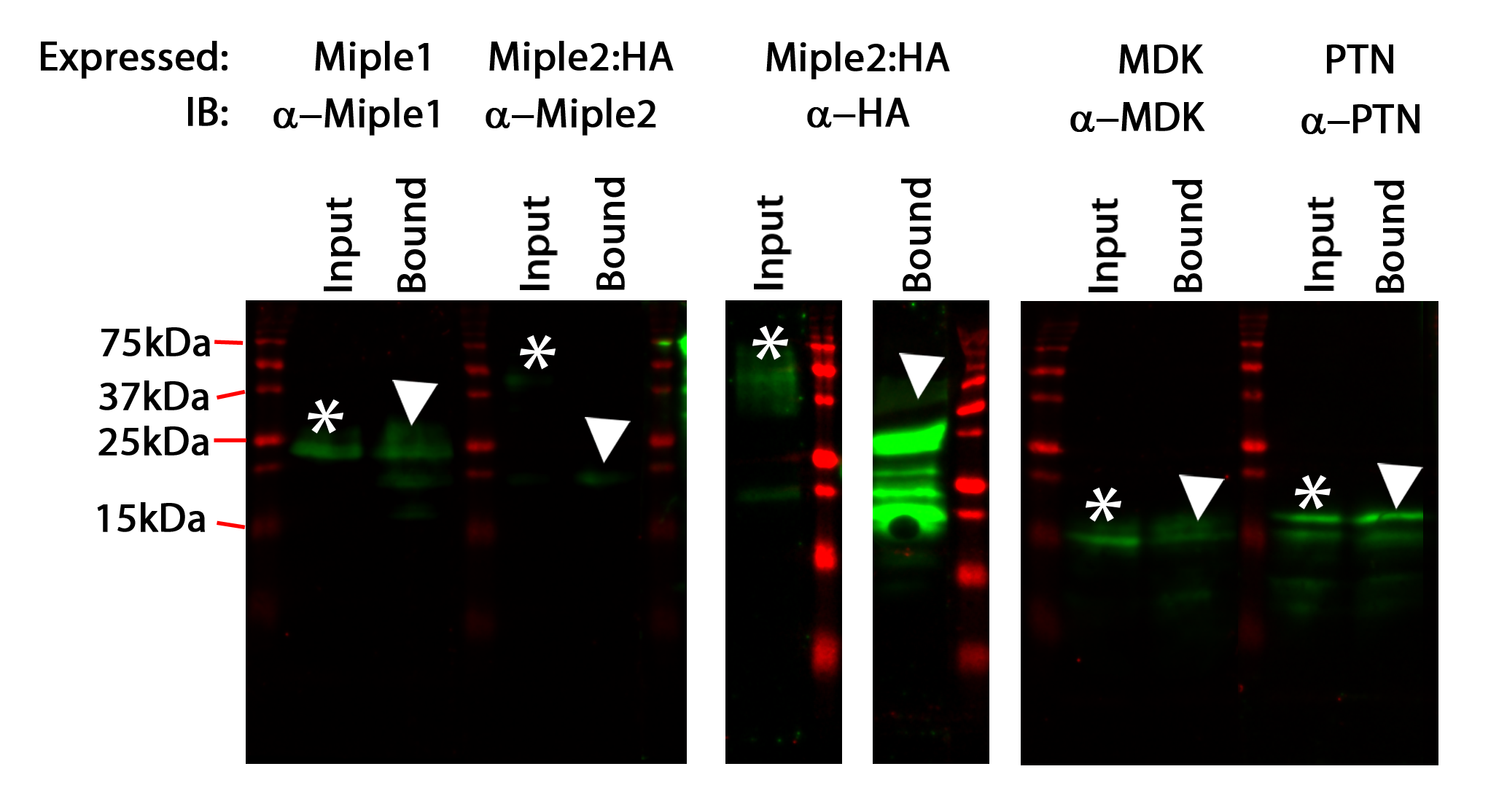

Supplement: Figure S3 — Miple proteins bind heparin in vitro . Western blot analysis of Miple proteins bound to heparin-agarose. Miple1 protein was detected in the conditioned media fraction (20 µl) (input, *) as well as in the heparin-agarose eluted fraction (20 µl) (bound, arrowhead). Similar results were observed with Miple2-HA detected with either HA antibody or Miple2 antibody, although in the case of using anti-Miple2, a smaller, potentially degraded or processed Miple2 protein was detected (input, * and bound, arrowhead), while with anti-HA several smaller bands was detected, suggesting a degradation of bound Miple2 protein. The human MDK and human PTN proteins are clearly detected in conditioned media (input,*) as well as in the heparin eluate (bound, arrowhead). (TIF) [file pone.0112250.s003.tif]

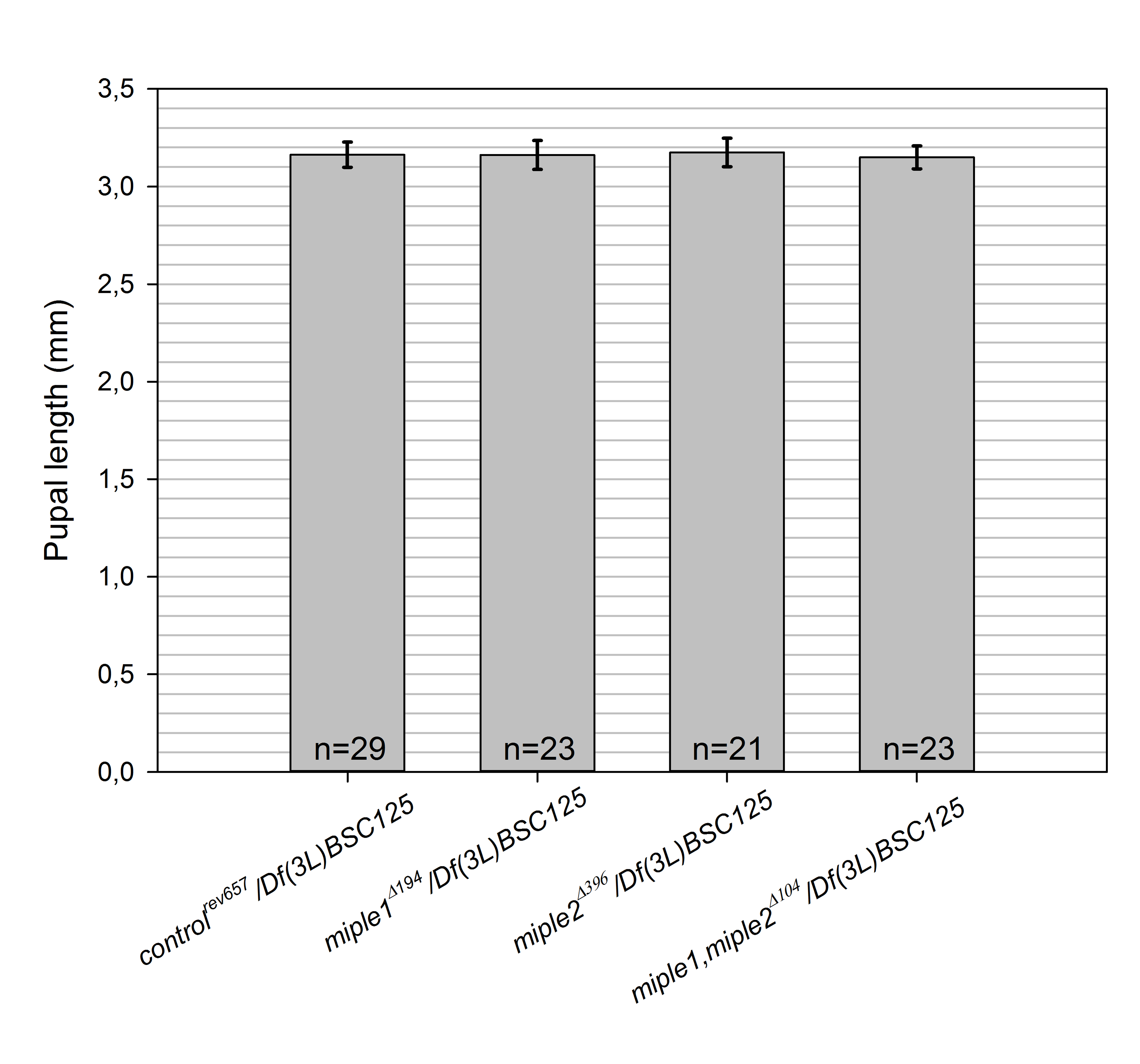

Supplement: Figure S4 — Loss of Miple does not affect pupal size. Progeny from maternal zygotic miple mutant females crossed to males bearing the deficiency Df(3L)BSC125, were measured at late pupal stages. Pupae length (in mm) of miple1Δ194/Df(3L)BSC125 and miple2Δ396/Df(3L)BSC125 single mutants as well as double deficient miple1,miple2Δ104/Df(3L)BSC125 were comparable to control (controlrev657/Df(3L)BSC125). All analysed pupae were confirmed as female and error bars denote S.E.M. (TIF) [file pone.0112250.s004.tif]

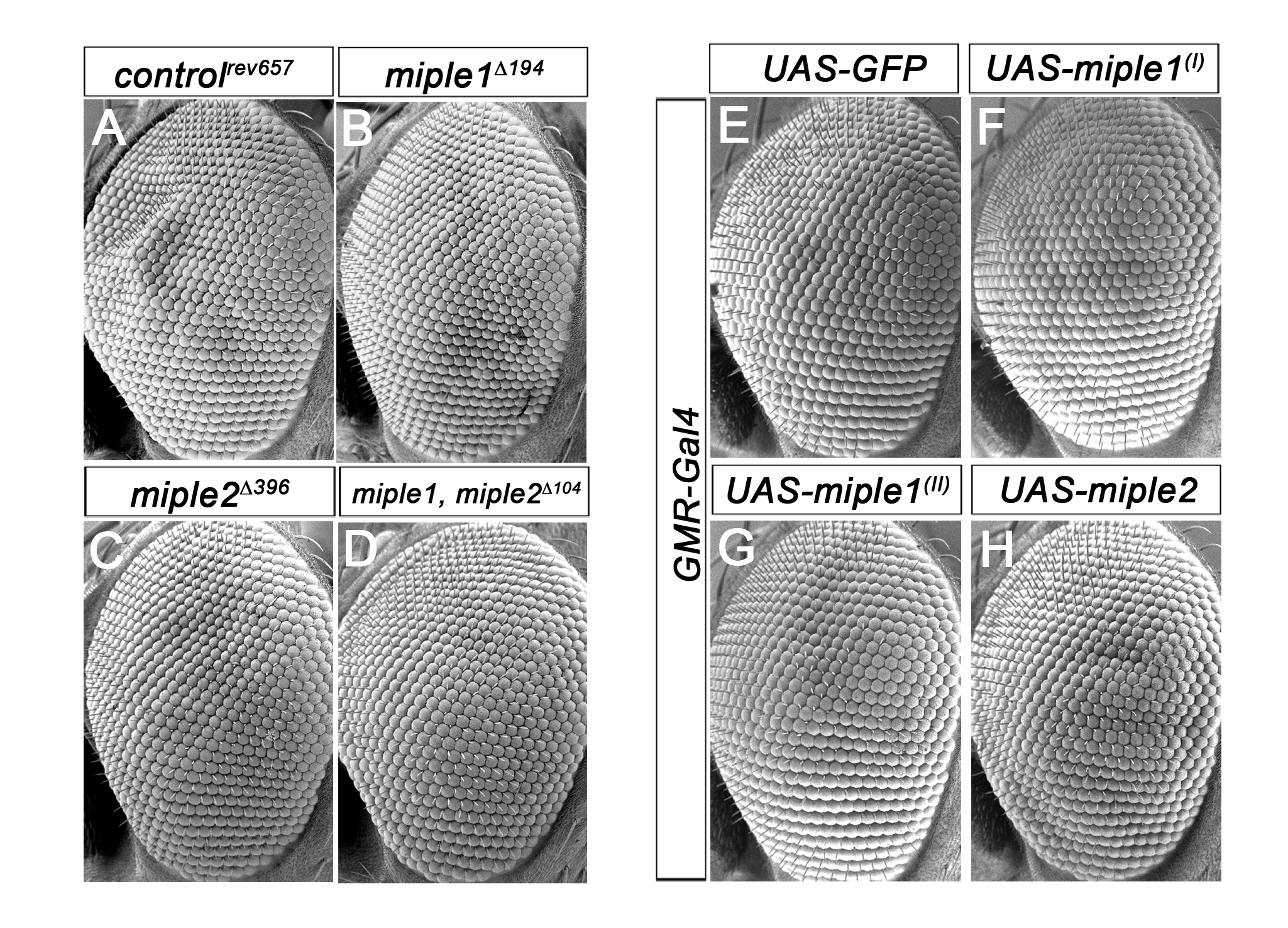

Supplement: Figure S6 — Neither loss or gain Miple proteins result in obvious developmental phenotypes. (A-H) Loss of Miple proteins or their over-expression, does not result in gross developmental defects, exemplified here by scanning EM of the Drosophila eye. No defects in ommatidal organization are observed in miple1Δ194/Df(3L)BSC125 (B), miple2Δ396/Df(3L)BSC125 (C) single mutant flies, or miple1,miple2Δ104/Df(3L)BSC125 (D) double mutant flies. Revertant controlrev 657 /Df(3L)BSC125 were employed as control (A). (E-H) Scanning EM photographs of adult eyes overexpressing GMR-Gal4 driven Miple1 protein employing two independent UAS-miple1 transgenic lines (F, G) and UAS-miple2 (H). No effect on eye morphology was observed when compared with controls expressing GFP (E). (TIF) [file pone.0112250.s006.tif]

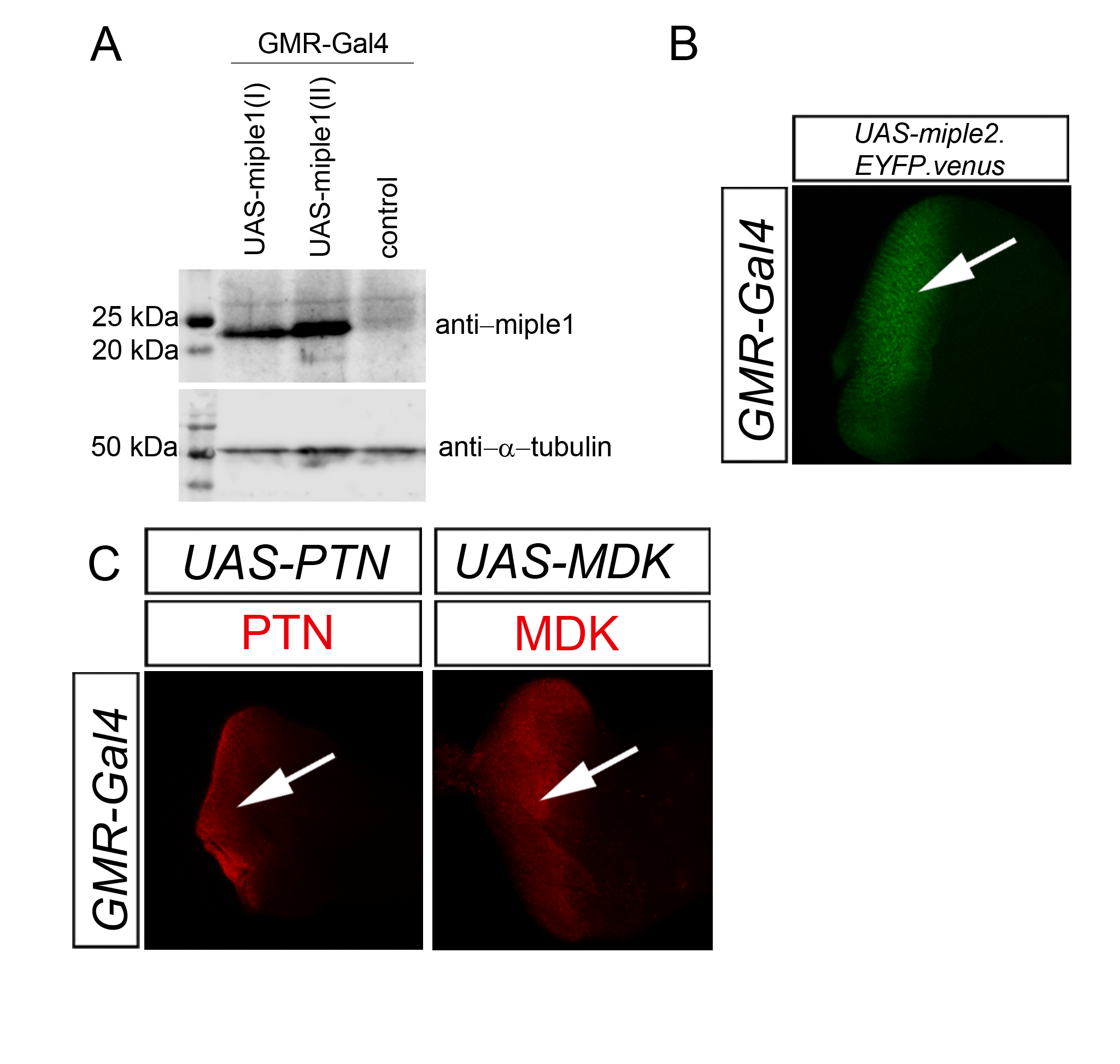

Supplement: Figure S7 — Confirmation of expression of Miple1 and Miple2 as well as human MDK and PTN transgenes. (A) To confirm expression of UAS-miple1 transgenes, total protein from heads of newly hatched adult flies overexpressing Miple1 by the GMR-Gal4 was analysed by western blotting. Miple1 can be detected for two independent transgene insertions but not in the negative control, showing that overexpressed and not endogenous Miple1 protein is detected. (B) Expression of Miple2.YFP.venus protein driven by GMR-Gal4 can clearly be detected in the larval eye disc (C) Expression of human PTN and human MDK protein in larval eye discs expressed by GMR-Gal4 was confirmed by immunohistochemistry and show that these transgenes are functional. (TIF) [file pone.0112250.s007.tif]

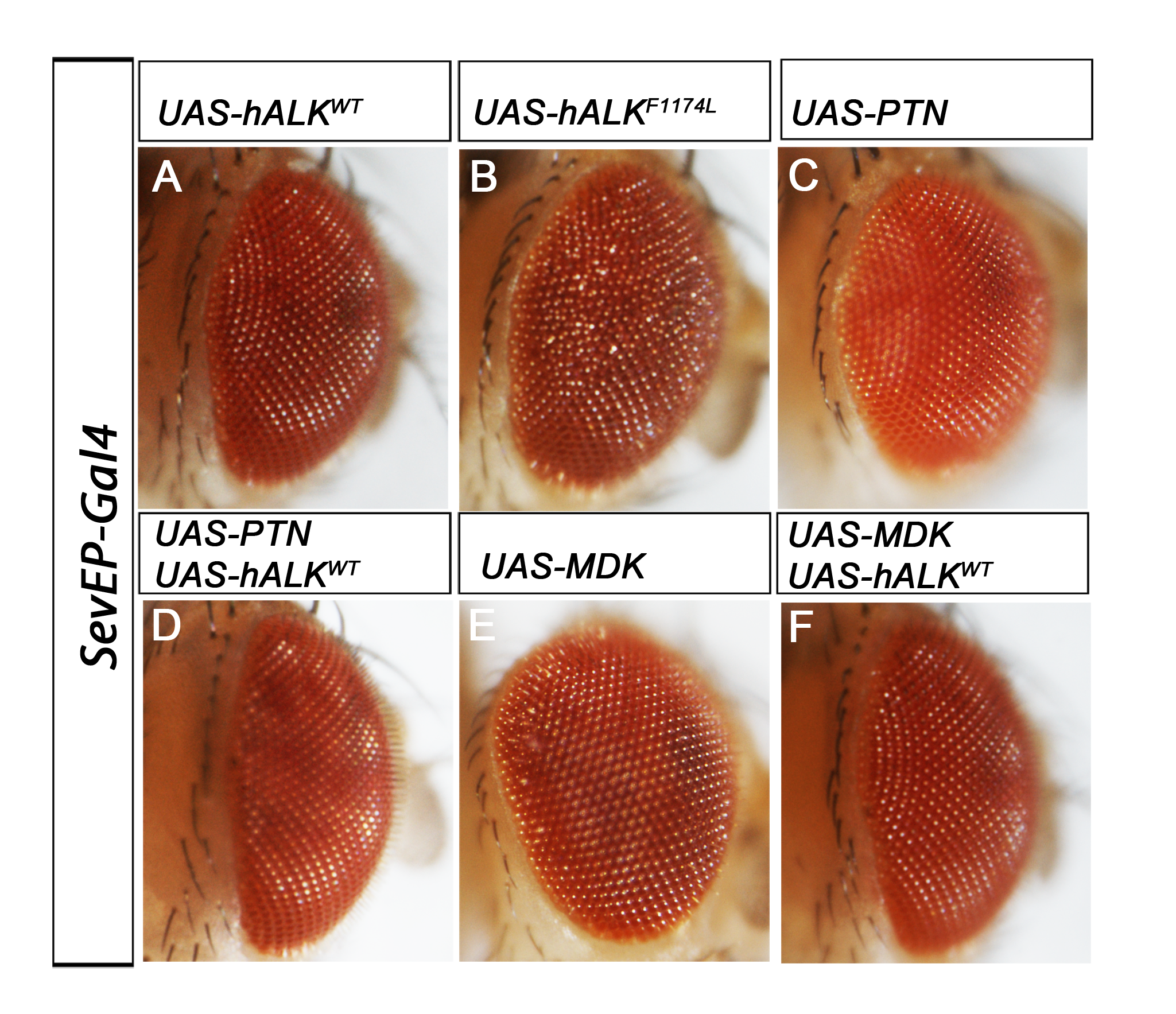

Supplement: Figure S8 — sevEP-Gal4 driven expression of human Miple homologues MDK and PTN with hALK does not affect eye morphology. (A-F) Ectopic expression of human PTN (C) and MDK (E) alone and co-expression with wild-type human ALK (D, F) in the in developing eye using sevEP-Gal4 does not affect adult eye morphology. As positive control expression of constitutively active hALK F1174L gain-of-function mutation (observed in human neuroblastoma) was employed (B). (TIF) [file pone.0112250.s008.tif]

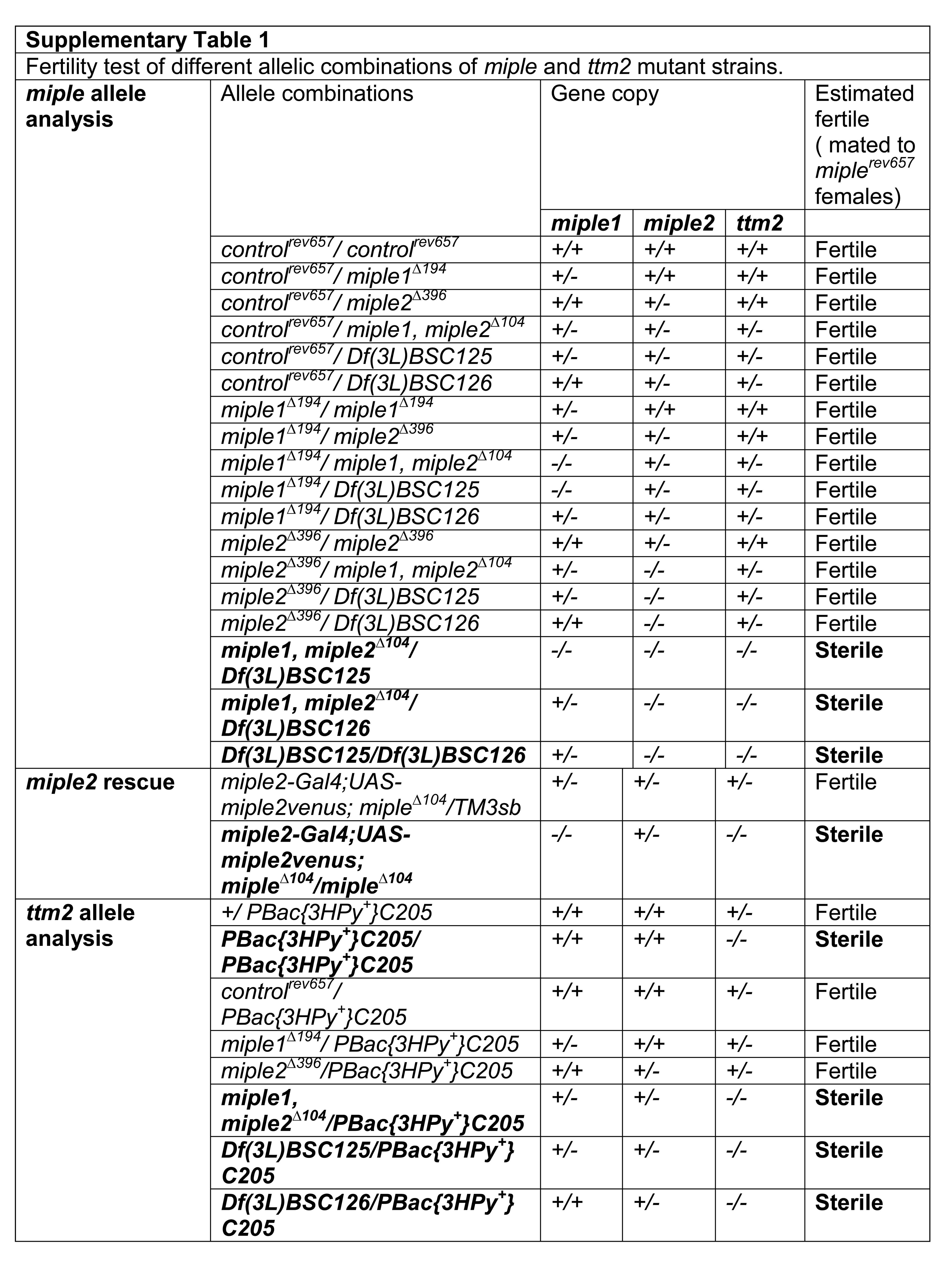

Supplement: Table S1 — Fertility test of different allelic combinations of miple and ttm2 mutant strains. Males with the indicated allelic combinations were generated and crossed to female controlrev 657 and scored for fertility measured by the presence of F1 1st Instar larvae. Expression of miple2 employing miple2Gal4>UAS-miple2 in miple1,mipleψΔ∼iψ mutant background fails to rescue the male sterile phenotype. All allelic combinations that produce sterile males contain homozygous deletion of the ttm2 gene. Additionally a P-element insertion PBac{3PHy+}C205 upstream of ttm2 generates heterozygous fertile but homozygous sterile males and produces male sterility in transheterozygous combination with the three strains that carry deletions covering ttm2 (miple1,mipleψΔ∼iψ, Df(3L)BSC125 and Df(3L)BSC126). (TIF) [file pone.0112250.s009.tif]

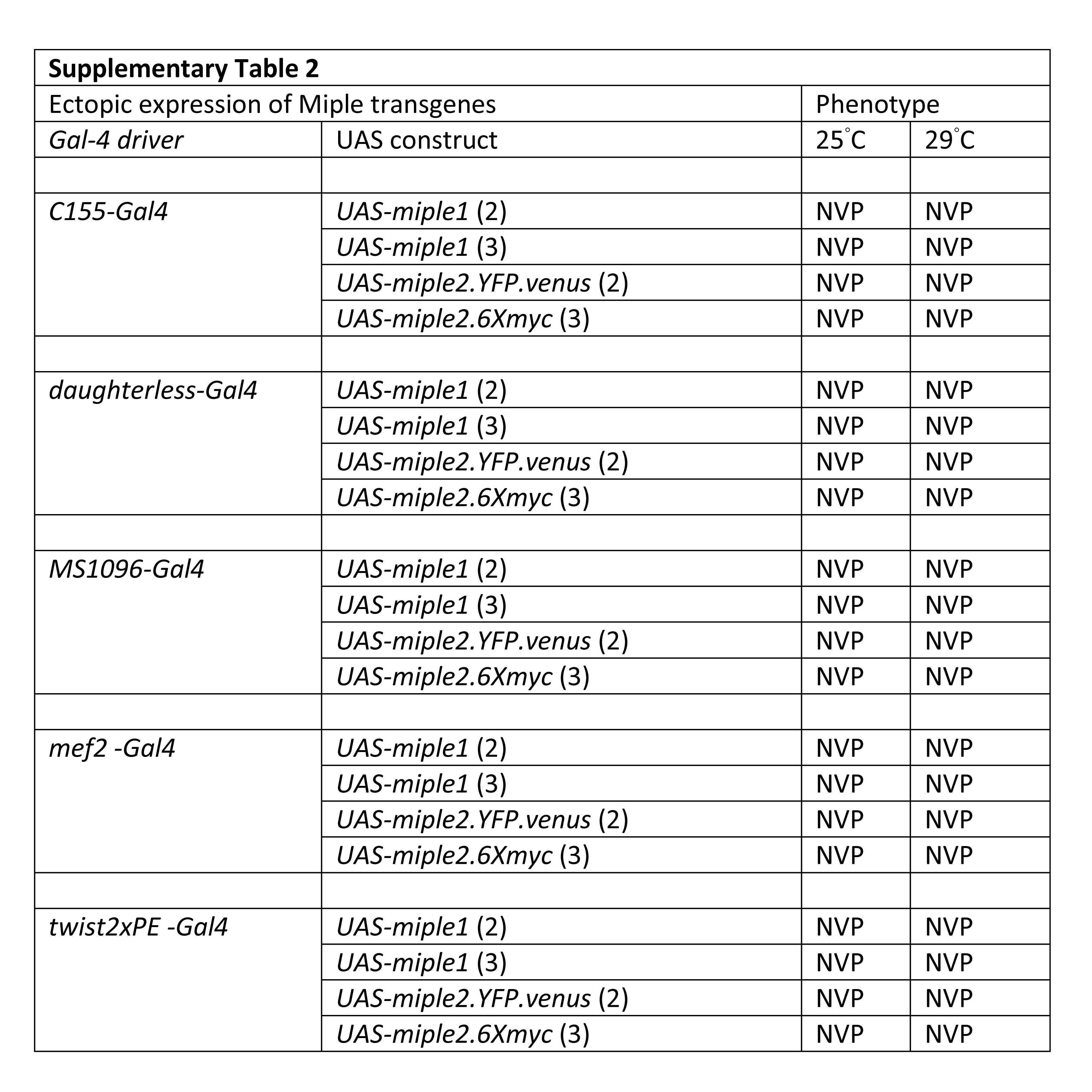

Supplement: Table S2 — Ectopic over-expression small screen of Miple transgenes. A panel of various Gal4 strains was crossed to several independent UAS-miple transgenes at two different temperatures (25 and 29 degrees). The progeny from the indicated crosses was scored for phenotype after hatching. All tested transgenes in combination with all tested Gal4 reveal no obvious phenotypes, in table scored as NVP (No Visible Phenotype) indicating that over-expression of these transgenes is not harmful for the fly. (TIF) [file pone.0112250.s010.tif]
